# Signal strength of STING activation determines cytokine plasticity and cell death in human monocytes

**DOI:** 10.1038/s41598-022-20519-7

**Published:** 2022-10-24

**Authors:** Dieter Kabelitz, Michal Zarobkiewicz, Michelle Heib, Ruben Serrano, Monika Kunz, Guranda Chitadze, Dieter Adam, Christian Peters

**Affiliations:** 1 Institute of Immunology, Christian-Albrechts University Kiel, Arnold-Heller-Str. 3, Building U30, 24105 Kiel, Germany; 2 Unit for Hematological Diagnostics, Department of Internal Medicine II, University Hospital Schleswig-Holstein, 24105 Kiel, Germany; 3 Present Address: Department of Clinical Immunology, Medical University of Lublin, 20-093 Lublin, Poland; 4 Present Address: Institute of Immunology, Medical University Hannover, 30625 Hannover, Germany

**Keywords:** Innate immunity, Pattern recognition receptors, Immunology, Innate immune cells, Monocytes and macrophages

## Abstract

The cyclic GMP-AMP synthase (cGAS)/stimulator of interferon genes (STING) pathway is a cytosolic sensor of microbial and host-derived DNA and plays a key role in innate immunity. Activation of STING by cyclic dinucleotide (CDN) ligands in human monocytes induces a type I interferon response and production of pro-inflammatory cytokines associated with the induction of massive cell death. In this study we have re-evaluated the effect of signal strength of STING activation on the cytokine plasticity of human monocytes. CDN (2′3′c-GAMP) and non-CDN (diABZI, MSA-2) STING ligands in the range of EC_50_ concentrations (15 μM 2′3′c-GAMP, 100 nM diABZI, 25 μM MSA-2) induced IFN-β, IP-10, and large amounts of IL-1β and TNF-α, but no IL-10 or IL-19. Interestingly, LPS-induced production of IL-10 and IL-19 was abolished in the presence of diABZI or MSA-2, whereas IL-1β and TNF-α were not inhibited. Surprisingly, we observed that tenfold lower (MSA-2, i.e. 2.5 μM) or 100-fold lower (diABZI, i.e. 1 nM) concentrations strongly stimulated secretion of anti-inflammatory IL-10 and IL-19, but little of IL-1β and TNF-α. Induction of IL-10 was associated with up-regulation of *PRDM1* (Blimp-1). While cytokine secretion stimulated by the higher concentrations was accompanied by apoptosis as shown by cleavage of caspase-3 and PARP-1, the low concentrations did not trigger overt cell death yet induced cleavage of gasdermin-D. Our results reveal a previously unrecognized plasticity of human monocytes in their signal strength-dependent production of pro- versus anti-inflammatory cytokines upon STING activation.

## Introduction

The cyclic GMP-AMP synthase (cGAS)-stimulator of interferon genes (STING) pathway is a powerful cytosolic sensor of microbial and host-derived DNA species and plays a key role in regulating innate immune responses in the context of infection, cell stress and tissue damage^1^. cGAS senses cytoplasmic double-stranded DNA and catalyzes the production of cyclic dinucleotides (CDN) like 2′3′ cyclic GMP-AMP dinucleotide (cGAMP) which then bind to STING dimers localized at the ER membrane^2^. STING recruits TANK-binding kinase-1 (TBK1), thereby promoting TBK1 phosphorylation and recruitment and subsequent phosphorylation of interferon (IFN) regulatory factor 3 (IRF3). Following translocation to the nucleus, this cascade initiates the expression of type I IFN genes, IFN-stimulated genes, and several chemokines. Activation of STING also leads to the activation of the inflammasome and NF-κB, and thereby to the production of pro-inflammatory cytokines^3,4^. STING is widely expressed in immune cells including T cells, B cells and myeloid cells, but the cellular responses to STING activation vary substantially^5–9^. Notably, STING activation is frequently associated with the induction of various forms of cell death including apoptosis, pyroptosis and lysosomal cell death^5,6,9–12^. In human monocytes, activation of STING activates the inflammasome in a NLRP3-dependent manner, independently of a priming step required for canonical NLRP3 activation^13^, leading to caspase-1 activation and release of IL-1β and IL-18. Upstream of the NLRP3-inflammasome activation, and in addition to triggering the IFN type I response, STING activation also triggers a lysosomal cell death program in monocytic cells^9^.

Without use of transfection reagents, the cellular uptake of exogenous cGAMP and similar CDN STING ligands varies between cell types, and SLC19A1 and LRRC8A have been identified as cell type-specific transporters of CDN across cell membranes^14–16^. Furthermore, an alternatively spliced STING isoform localized in the cytoplasmic membrane was recently shown to directly sense extracellular cGAMP^17^. Taken together, these cell type-specific variables add to the complexity when considering the use of cGAMP and related CDN as adjuvants in cancer therapy^18^. In this respect, the recent discovery of highly selective non-CDN STING ligands has opened new perspectives for therapeutic application of STING ligands to activate innate immunity and co-stimulate adaptive immunity^19–21^. Such STING ligands can directly enter into cells and have potent anti-tumor activity^19,21,22^, but also potently protect against SARS-CoV-2 infection^23,24^.

Non-CDN STING ligands diABZI and MSA-2 are highly potent and induce TBK1/IRF3 phosphorylation and type I IFN and inflammatory cytokine secretion in human peripheral blood mononuclear cells (PBMC) at reported EC_50_ of 130 nM (diABZI) and 8.3 µM (MSA-2)^19,21^. Such results suggest that activation of the cGAS/STING pathway can only result in induction of pro-inflammatory cytokines and thus promote inflammation. In this study, however, we demonstrate a concentration-dependent biphasic plasticity of human monocytes in response to STING activation by diABZI and MSA-2. As expected, concentrations in the EC_50_ range induced TBK1 phosphorylation, IFN-β, IP-10, IL-1β and TNF-α but no IL-10 secretion, whereas two-log (diABZI) and one-log (MSA-2) lower concentrations strongly induced the secretion of anti-inflammatory cytokines IL-10 and IL-19 but very little of pro-inflammatory cytokines. Our results provide important new insights into the signal strength-dependent regulation of monocyte plasticity by STING ligands which are relevant when applying STING ligands therapeutically.

## Materials and methods

All methods were carried out in accordance with relevant regulations and guidelines.

### Monocytic cells and cell cultures

Leukocyte concentrates obtained from healthy adult blood donors were kindly provided by the Dept. of Transfusion Medicine, University Hospital Schleswig-Holstein (UKSH) Campus Kiel. Informed consent was provided by all blood donors, and the use of leukocytes for research was approved by the Ethics Committee of the Medical Faculty (code D405/10). Monocytes were isolated from Ficoll-Hypaque separated PBMC by negative magnetic selection using the Pan Monocyte (order no. 130-096-537) or the Classical Monocyte (order no. 130-117-337) kits from Miltenyi Biotec (Bergisch Gladbach, Germany), or the EasySep Human Monocyte kit from StemCell Technologies (Cologne, Germany) according to the manufacturer’s protocols. Monocytes were differentiated for 6 days in the presence of 50 ng/mL M-CSF (ImmunoTools, Friesoythe, Germany). Fresh M-CSF was added after 4 days. Freshly isolated monocytes and M-CSF-differentiated macrophages were cultured at 10^5^ cells per well in round-bottom 96-well microtiter plates in RPMI 1640 medium (GIBCO) supplemented with 100 U/mL penicillin, 100 µg/mL streptomycin and 10% heat-inactivated low endotoxin fetal bovine serum (Bio&Sell, Feucht, Germany). In some experiments, monensin (3 μM) was added to monocytes cultures for 4 h before processing for intracellular IL-10 staining.

### STING/TLR ligands and inhibitors

The following ligands and inhibitors were obtained from Invivogen (Toulouse, France): Ultrapure LPS (TLR4); STING ligands: 2′3′-cGAMP (cGAMP), 2′3′-cGAMP control (cGAMP ctr); TBK1 inhibitor BX795. TLR8 agonist Motolimod (VTX-2337) was purchased from Sellekchem (Houston, TX, USA). STING ligands diABZI and MSA-2 were obtained from MedChemExpress (Biozol, Eching, Germany). Stock solutions of all ligands and inhibitors were prepared according to the manufacturer’s instructions. Final concentrations of DMSO (where appropriate) never exceeded 0.1%; corresponding DMSO controls did not have any effect.

### Quantification of cytokines

DuoSet sandwich ELISAs for detection of human IL-10, IL-19, IL-1β, TNF-α, IFN-β, IP-10, IL-37, IL-12p70 were obtained from R&D Systems (BioTechne, Wiesbaden, Germany). All measurements were from two independent biological replicates. The assays were performed according to the instructions of the manufacturer.

### Flow cytometry

The following mAb were obtained from BD Biosciences (Heidelberg, Germany): anti-CD14-FITC/APC (clone MoP9), anti-IL-10-PE/FITC (JES3-19F1), anti-CD16-PE (clone B73.1). Anti-CD163-PE/FITC (clone GHI/61.1) was purchased form Miltenyi Biotech, anti-STING-AF488 (clone 723505) from R&D Systems, and anti-CD206-FITC (clone 15-2) from BioLegend (Amsterdam, The Netherlands). Rat monoclonal anti-p-TBK-1-PE (clone D52C2) and control monoclonal rat-Ig-PE were obtained from Cell Signaling Technology Europe (Frankfurt, Germany). Cell death was measured by combined annexin V-FITC (Mabtag, Friesoythe, Germany) and propidium iodide (PI) staining. For cell surface staining, cells were washed, stained for 20 min on ice with mAb, washed twice, and resuspended in 1% paraformaldehyde. For intracellular staining, cells were permeabilized in Cytofix/Cytoperm buffer (BD Biosciences) before staining with fluorochrome-conjugated mAb. All analyses were measured on a FACS-Canto or LSR-Fortessa cytometer (BD Biosciences), using DIVA (Data-Interpolating Variational Analysis) for acquisition and FlowJo v10.6.1 (FlowJo, Ashland, OR, USA) for data analysis.

### Western blot

2 to 4 × 10^6^ purified monocytes were incubated at 37 °C for 4 to 7 h with STING ligands at concentrations as indicated under results. Cells were lysed at 4 °C in TNE buffer (50 mM Tris pH 8.0, 150 mM NaCl, 1% (v/v) NP-40, 3 mM EDTA, 1 mM sodium orthovanadate, 5 mM sodium fluoride) containing 10 µg/mL Roche cOmplete protease inhibitor (Merck, Darmstadt, Germany). 20 μg of protein per lane were separated by SDS–polyacrylamide gel electrophoresis. After transfer onto nitrocellulose membranes, reactive proteins were detected with antibodies specific for phospho-TBK1 (#5483, TBK1 (#3504), cleaved caspase-3 (#9661), cathepsin-D (#2284), PARP-1(#9542), gasdermin D (#39754) from Cell Signaling Technology, STING (#675902) from BioLegend, β-actin (#A1978) from Sigma-Aldrich (Taufkirchen, Germany). Clarity Western ECL (Bio-Rad, Feldkirchen, Germany) or LumiGLO ECL substrate (Cell Signaling Technology) were used for chemiluminescence detection using Hyper Film (GE Healthcare Buckinghamshire, UK). Equal loading and transfer efficiency were routinely verified for all Western Blots by Ponceau S staining and re-probing the membranes with anti-β-actin antibody.

### Quantitative RT-PCR

RNA was isolated using the RNeasy mini-Kit (Qiagen, Hilden, Germany). The reverse-transcription of 250 ng mRNA was performed at 37 °C for 1 h using 3 µg random hexamer primers (Thermo Fisher Scientific, Waltham, MA, USA), 25 nmol per dNTP (Bioline, London, UK), 200 IU M-MLV Reverse Transcriptase (Promega, Madison, WI, USA) and M-MLV Reverse Transcriptase Reaction Buffer (Promega) in a total volume of 20 µL in the presence of 20 IU RNasin Plus RNase Inhibitor (Promega). The gene expression levels of *IL10* and *PRDM1*_tv2 were quantified by RT-PCR in relation to three different house-keeping genes (*G6PDH*, *HuPo*, *RPII*). To this end, 0.8 µL cDNA and 10 pmol of a forward and a reverse primer were added to 10 µL of a 2 × concentrated reaction mix PowerUp SYBR Green Master Mix (Thermo Fisher Scientific) in a final volume of 20 µL. The quantitative RT-PCR was performed in 3 steps, with 15 s at 95 °C, 15 s at 55 °C and 60 s at 70 °C. The gene expression was measured on a QuantStudio 5 Real-Time PCR System (QS5 0.2ML QPCR SYSTEM, Thermo Fisher Scientific). The quality of the PCR-products was controlled by subsequent melt curve analysis. Specific primers for RT-PCR of *IL10* [IL10_fw (TgCCTTCAgCAgAgTgAAgA); IL10_rev (gCAACCCAggTAACCCTTAAA)] and *PRDM1* [PRDM1-tv2_fw (gTggTgggTTAATCggTTTg); PRDM1-tv2_rev (gAAgCTCCCCTCTggAATAgA)] were purchased from TIB MolBiol (Berlin, Germany). RT-PCR data analysis was performed using QuantStudio Design & Analysis Software v.1.5.1.

### Statistical analysis

Results are depicted as box and whisker plots where the horizontal bar represents the median and the box below and above the second and third quartile, respectively. Whiskers represent values lying outside of the boxes. The Shapiro–Wilk normality test was used to determine the normal distribution of experimental values. In case of non-normal distribution, the Wilcoxon signed rank test was applied for statistical comparisons, otherwise the paired Student’s t-test was used. All analyses were done with “R” using R-Studio V. 1.1.463 software together with the ggpubr package and ggplot2 and ggbeeswarm packages for scatter plot figure visualization. Levels of significance were set as *p < 0.05, **p < 0.01, ***p < 0.001.

### Ethical approval

This study was performed in line with the principles of the Declaration of Helsinki. Approval was granted by the Ethics Committee of the Medical Faculty of Kiel University (code D405/10).

## Results

### STING ligands stimulate IL-1β/TNF-α but no IL-10/IL-19 secretion in human monocytes

**Figure 1:**
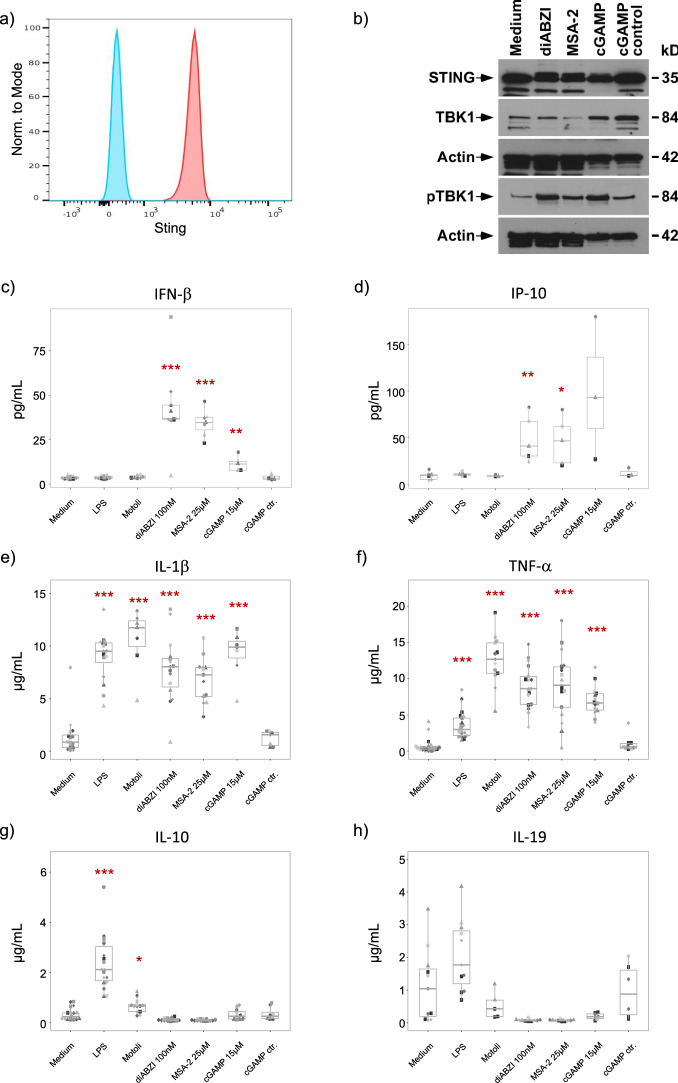
Responsiveness of monocytes to STING ligands. Purified monocytes were stimulated with 100 ng/mL LPS, 0.5 µg/mL Motolimod,100 nM diABZI, 25 µM MSA-2, or 15 µM cGAMP/cGAMP control as indicated. (**a**) STING expression in monocytes was analyzed by flow cytometry. Blue: FMO control, red: anti-STING-AF488. One representative out of five experiments. (**b**) Western blot analysis of monocyte lysates after 4 h stimulation. (**c**–**h**) Cytokines were quantified by ELISA in supernatants after 24 h. Each donor is identified by identical symbol and grey tone. (**c**) IFN-β (n = 5–9). (**d**) IP-10 (n = 3–5). (**e**) IL-1β (n = 7–18). (**f**) TNF-α (n = 10–22). (**g**) IL-10 (n = 8–20). (**h**) IL-19 (n = 5–12). Statistical significance was calculated for each group by comparing stimulatory conditions with medium by paired Student’s t-test or Wilcoxon signed rank test as appropriate. Red asterisks indicate the level of significance between stimulated and unstimulated (medium) samples. *p < 0.05, **p < 0.01, ***p < 0.001.

**Figure 2:**
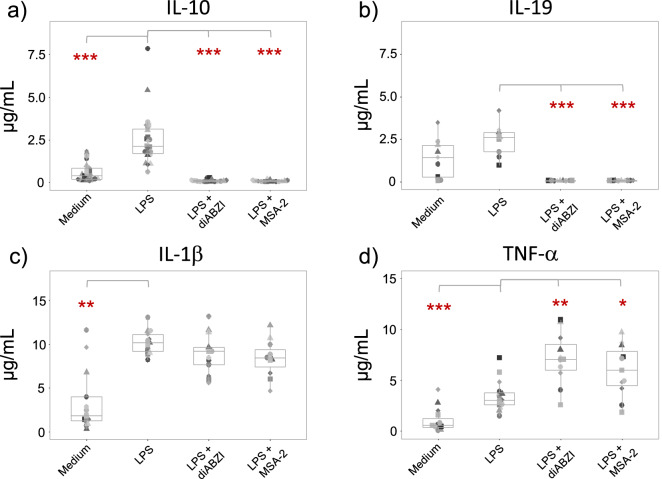
STING ligands suppress LPS-induced secretion of anti-inflammatory but not pro-inflammatory cytokines. Purified monocytes were cultured in medium only or were stimulated with 100 ng/mL LPS in the absence or presence of 100 nM diABZI or 25 µM MSA-2 as indicated. Supernatants were collected after 24 h, and cytokines were quantified by ELISA. (**a**) IL-10 (n = 15–23). (**b**) IL-19 (n = 9). (**c**) IL-1β (n = 11–13). (**d**) TNF-α (n = 11–15). Statistical significance was calculated between medium and LPS and for LPS + diABZI and LPS + MSA-2 groups in comparison to LPS only as indicated by the horizontal bars. Wilcoxon signed rank test. was used. *p < 0.05, **p < 0.01, ***p < 0.001.

Purified monocytes expressed STING as shown by flow cytometry (Fig. 1a) and Western blot analysis (Fig. 1b). Monocytes were stimulated for 20 h with CDN (15 µM cGAMP) and novel non-CDN (100 nM diABZI, 25 µM MSA-2) STING ligands in the range of reported EC_50_ concentrations, and supernatants were collected for measurement of various cytokines by ELISA. As expected, STING ligands activated the type I IFN signaling pathway in monocytes as revealed by TBK1 phosphorylation (Fig. 1b) and the induction of IFN-β (Fig. 1c) and IP-10 (Fig. 1d). Remarkably, non-CDN ligands diABZI and MSA-2 were more potent IFN-β inducers in monocytes than cGAMP. As expected, both TLR4 ligand LPS as well as TLR8 ligand Motolimod failed to stimulate IFN-β or IP-10 (Fig. 1c,d). All analyzed ligands except the inactive cGAMP control potently induced the secretion of IL-1β (Fig. 1e) and TNF-α (Fig. 1f) confirming that the cGAS/STING pathway also triggers the production of pro-inflammatory cytokines^9,25^. Interestingly, however, the standard concentrations of STING ligands shown above to efficiently activate the type I IFN pathway (Fig. 1b–d) and pro-inflammatory cytokines (Fig. 1e,f) did not elicit any secretion of anti-inflammatory IL-10 (Fig. 1g) or IL-19 (Fig. 1h). As shown, both cytokines were induced by LPS, while Motolimod was quite inefficient, in line with the known low activity of TLR8 to trigger IL-10 production^26^. Only very low levels of IL-12p70 and IL-37 were measured in supernatants, independently of the specific ligand (not shown). Together, these results indicate that the commonly used concentrations of STING ligands activate the STING/type I IFN pathway and pro-inflammatory cytokines in human monocytes but do not induce anti-inflammatory cytokines. Moreover, we observed that both diABZI and MSA-2 not only did not induce IL-10 and IL-19 (Fig. 1g,h), but in fact completely suppressed the secretion of IL-10 (Fig. 2a) and IL-19 (Fig. 2b) in monocytes stimulated by LPS when added together with LPS. This effect was specific for IL-10 and IL-19, as IL-1β secretion in response to LPS was only moderately reduced in presence of STING ligands (Fig. 2c), whereas secretion of TNF-α in response to LPS was in fact significantly co-stimulated in the presence of STING ligands (Fig. 2d).

### Concentration-dependent biphasic pattern of pro- versus anti-inflammatory cytokine induction

**Figure 3:**
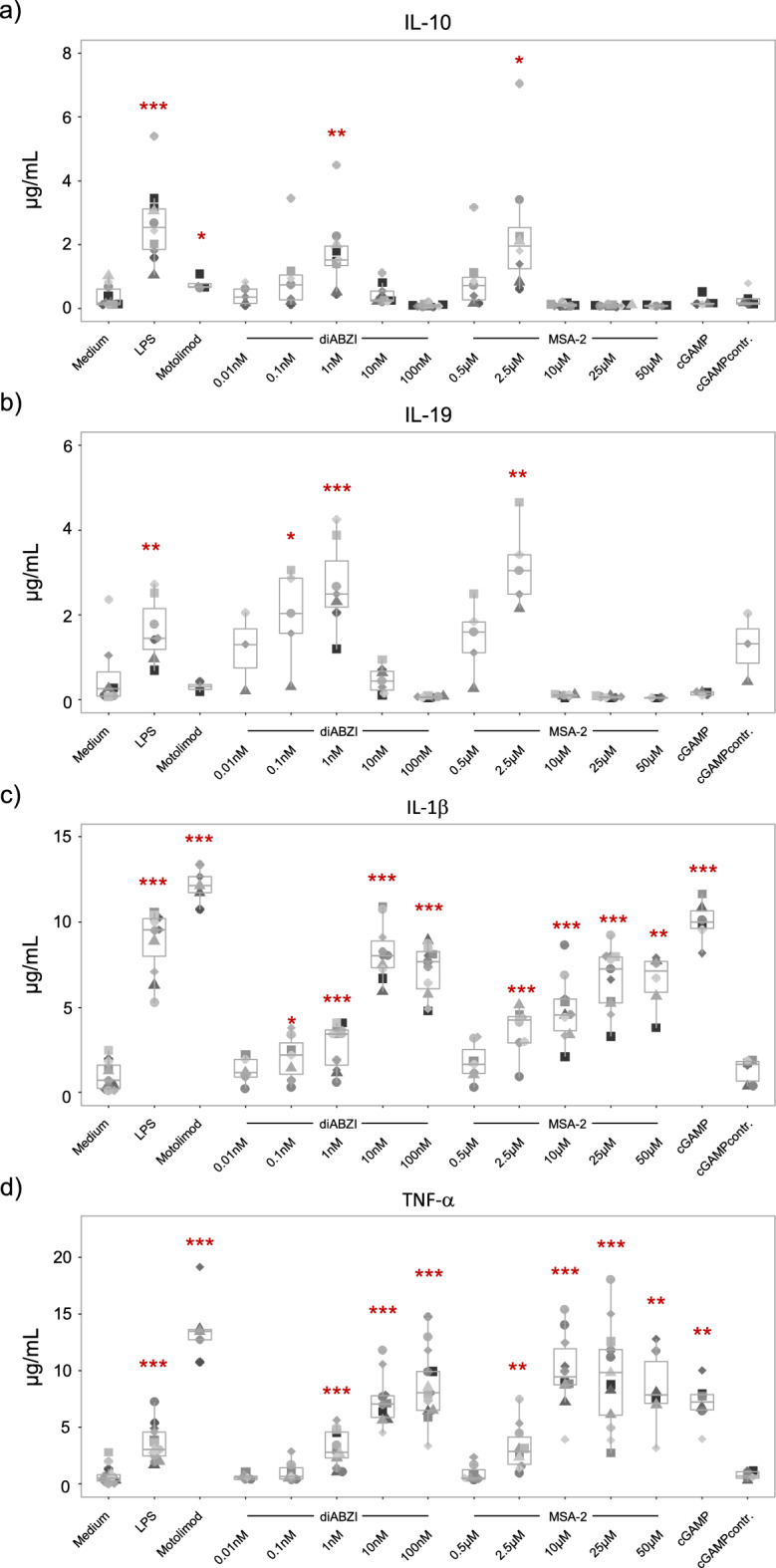
Biphasic pattern of cytokine induction in monocytes. Purified monocytes were cultured in medium only or were stimulated with 100 ng/mL LPS, 0.5 µg/mL Motolimod, 0.01–100 nM diABZI, 0.5–50 µM MSA-2, or 15 µM cGAMP/cGAMP control as indicated. Cytokines were quantified by ELISA in supernatants after 24 h. Each donor is identified by identical symbol and grey tone. (**a**) IL-10 (n = 4–10). (**b**) IL-19 (n = 3–7). (**c**) IL-1β (n = 5–11). (**d**) TNF-α (n = 5–13). Statistical significance was calculated for each group by comparing stimulatory conditions with medium using paired Student’s test. Red asterisks indicate the level of significance between stimulated and unstimulated (medium) samples. *p < 0.05, **p < 0.01, ***p < 0.001.

In a next step, we repeated the stimulation of purified monocytes using lower concentrations of non-CDN STING ligands. For comparison, we used a fixed concentration of CDN cGAMP (15 µM) and TLR ligands LPS (100 ng/mL) and Motolimod (0.5 µg/mL) as before. Supernatants were collected after 24 h for measurement of IL-10, IL-19, IL-1β and TNF-α (Fig. 3). As already shown above, the standard concentrations in the EC_50_ range of diABZI (100 nM) and MSA-2 (10–50 µM) did not stimulate any IL-10 (Fig. 3a) or IL-19 (Fig. 3b) release but strongly induced IL-1β (Fig. 3c) and TNF-α (Fig. 3d). Quite surprisingly, however, lower concentrations of diABZI (0.1–1 nM) and MSA-2 (0.5–2.5 µM) potently stimulated the secretion of both IL-10 (Fig. 3a) and IL-19 (Fig. 3b) but very little if any IL-1β (Fig. 3c) or TNF-α (Fig. 3d). The dose titrations shown in Fig. 3 a,b indicate optimal induction of IL-10 and IL-19 by 1 nM diABZI and 2.5 µM MSA-2, respectively, 100-fold and tenfold lower concentrations than the reported EC_50_ of the two non-CDN ligands (19,21). In view of the known capacity of the cGAS/STING pathway to initiate various forms of cell death in human monocytes, we analyzed morphological changes of purified monocytes in response to titrated concentrations of STING ligands by microscopic inspection (Suppl. Fig. S1). In untreated medium controls, the bottom of wells was filled with viable monocytes, whereas cells had largely disappeared in the presence of the regular STING ligand concentrations 100 nM diABZI and 25 µM MSA-2. In the presence of IL-10 inducing concentrations (1 nM diABZI, 2.5 µM MSA-2), the formation of activation clusters was observed (Suppl. Fig. S1).

### Characterization of IL-10 producing monocytes

**Figure 4:**
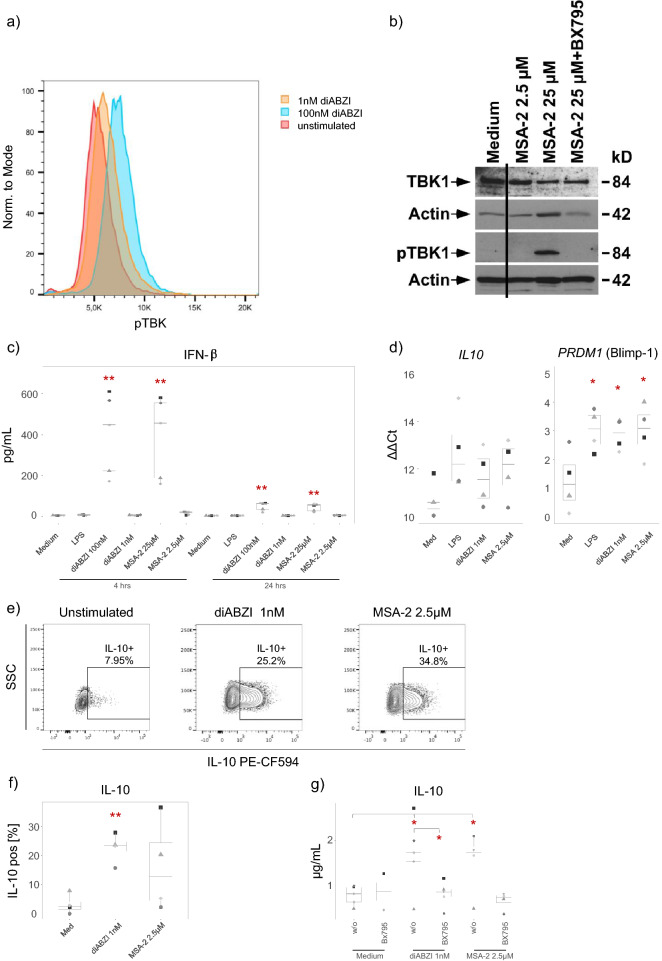
Characterization of IL-10 producing monocytes. Purified monocytes were stimulated with low (1 nM diABZI, 2.5 µM MSA-2) or regular (100 nM diABZI, 25 µM MSA-2) concentrations of STING ligands in the presence or absence of 1 µM TBK1 inhibitor BX795. (**a**) TBK1 phosphorylation in response to 1 or 100 nM diABZI was determined by flow cytometry after 60 min. One representative out of three experiments is shown. (**b**) TBK1 phosphorylation in response to 2.5 or 25 µM MSA-2 was revealed by Western blot after 4 h. (**c**) IFN-β in supernatants collected after 4 and 24 h was quantified by ELISA (n = 5). (**d**) qRT-PCR analysis of *IL10* and *PRDM1* (Blimp-1) in monocytes cultured for 8 h in medium or with 100 ng/mL LPS, 1 nM diABZI, or 2.5 µM MSA-2 (n = 4). ΔΔCt values refer to the change versus freshly isolated monocytes. (**e**,**f**) Detection of intracellular IL-10 by flow cytometry. Purified monocytes were stimulated with 1 nM diABZI or 2.5 µM MSA-2. Intracellular expression of IL-10 was determined after 24 h. (**e**) One representative is shown. A gate on IL-10 expressing cells was set in relation to the FMO control. (**f**) Summary of three experiments. (**g**) Monocytes were stimulated with 1 nM diABZI or 2.5 µM MSA-2 in the absence or presence of 1 µM BX795. IL-10 was quantified in supernatants collected after 24 h (n = 4–5, or 2 [medium with BX795]). Statistical significance was calculated by paired Student’s test. In (**c**,**d**,**f**), significance was calculated between stimulated and unstimulated (medium) groups. In (**g**), significance was calculated between diABZI/MSA-2 and medium as well as between diABZI in presence versus absence of BXT795 as indicated by horizontal bars. Red asterisks indicate the level of significance between stimulated and unstimulated (medium) samples. *p < 0.05, **p < 0.01.

Next we asked if the IL-10 induction by low dose STING ligands also involved activation of the type I IFN pathway. To this end, we analyzed TBK1 phosphorylation and IFN-β secretion of purified monocytes in response to “low” versus “normal” concentrations of diABZI and MSA-2. TBK1 phosphorylation was analyzed by flow cytometry (Fig. 4a) and Western blot (Fig. 4b). As shown in Fig. 4a, 100 nM diABZI induced a clear signal of TBK1 phosphorylation after 1 h, whereas only a minor shift of pTBK1 was observed with 1 nM diABZI in flow cytometry. Western blot analysis of TBK1 phosphorylation in response to MSA-2 revealed a clear signal at 25 µM (which was prevented by TBK1 inhibitor BX795) but no detectable TBK1 phosphorylation with the IL-10 inducing low concentration of 2.5 µM (Fig. 4b). TBK1 phosphorylation was also detectable by flow cytometry when using MSA-2, as was TBK1 phosphorylation detectable also by Western blot when using diABZI stimulation (not shown). Furthermore, neither 1 nM diABZI nor 2.5 µM MSA-2 triggered any IFN-β secretion at both early (4 h) and late (24 h) time points, whereas the regular concentrations of 100 nM diABZI and 25 µM MSA-2 strongly stimulated IFN-β secretion, notably already after 4 h (Fig. 4c). The induction of IL-10 secretion by low dose STING ligands was associated with up-regulation of *PRDM1* (Blimp-1) as revealed by qRT-PCR (Fig. 4d). In addition to ELISA, we also performed intracellular cytokine staining to identify IL-10 producing monocytes in response to low dose STING ligands. Representative dot plots are shown in Fig. 4e, and a summary of three independent experiments in Fig. 4f. Between 15 and 25% of monocytes expressed IL-10 in response to 1 nM diABZI, and between 5 and 35% of monocytes in response to 2.5 µM MSA-2. The IL-10 secretion induced by low dose STING ligands was prevented in the presence of TBK1 inhibitor BX795 (Fig. 4g).

The strong induction of IL-10 production by low concentrations of STING ligands (Figs. 3, 4) was observed with monocytes from 12 of 20 donors where spontaneous secretion in the absence of intentional activation was < 1 µg/mL. However, monocytes from some other donors displayed much higher spontaneous IL-10 secretion. In these instances, low dose diABZI and MSA-2 did not further stimulate IL-10 secretion, whereas 100 nM diABZI and 25 µM MSA-2 completely inhibited the spontaneous IL-10 secretion (Suppl. Fig. S2).

**Figure 5:**
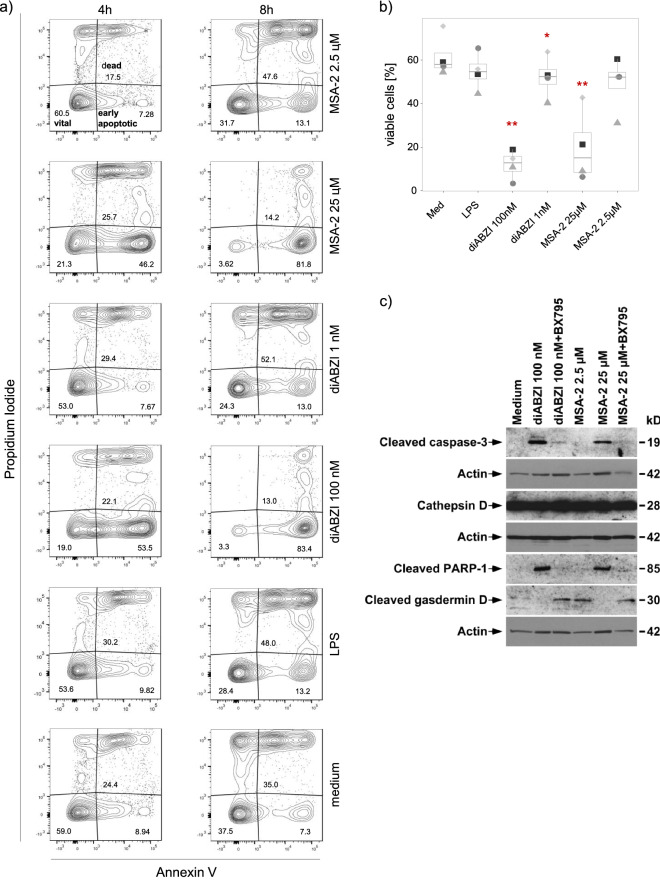
Cell death induction in monocytes by STING ligands. Purified monocytes were cultured in medium or were stimulated for 4 and 7 or 8 h with 100 ng/mL LPS or the indicated concentrations of STING ligands. Cell death was analyzed by flow cytometry following PI/annexin V staining and by Western blot analysis. (**a**) Contour plot of a representative experiment, left panel: 4 h, right panel: 8 h. (**b**) Summary of four experiments, displayed as proportion of viable (PI^−^/annexin V^−^) cells. Statistical significance was calculated by paired Student’s test. Red asterisks indicate the level of significance between stimulated and unstimulated (medium) samples. *p < 0.05, **p < 0.01. (**c**) Western blot analysis of death-associated proteins in monocytes after stimulation for 7 h with the indicated concentrations of STING ligands.

Microscopic inspection suggested differential levels of monocyte cell death induced by regular versus low concentrations of diABZI and MSA-2 (Suppl. Fig. S1). Therefore, cell death was further quantified by propidium iodide (PI)/annexin V staining using flow cytometry. As shown in a representative experiment in Fig. 5a, massive early apoptosis (Annexin V^+^/PI^−^) was induced already after 4 h by 100 nM diABZI (53.5%) and 25 µM MSA-2 (46.2%), whereas the lower concentrations of 1 nM diABZI and 2.5 µM MSA-2 as well as LPS did not affect cell death (Fig. 5a, left panel). At 8 h, increased level of cell death was already observed in medium, but again the lowest proportion of living cells (Annexin V^−^/PI^−^, lower left quadrant) was present in monocytes treated with 100 nM diABZI (3.3%) or 25 μM MSA-2 (3.6%) in comparison to medium (37.5%) (Fig. 5a, right panel). A summary of four experiments is presented in Fig. 5b, supporting the conclusion that regular but not low concentrations of non-CDN STING ligands strongly reduce the viability of human monocytes. Apoptotic cell death is characterized by the cleavage of caspase-3 and PARP-1. As shown in Fig. 5c, both 100 nM diABZI and 25 µM MSA-2 readily triggered the cleavage of caspase-3 and PARP-1 revealed by the appearance of the respective 19 kD (caspase-3) and 89 kD (PARP-1) fragments in Western blot. Cleavage of caspase-3 and PARP-1 was prevented in the presence of TBK1 inhibitor BX795. In contrast, cleavage of caspase-3 and PARP-1 was not observed with the low concentrations of 2.5 µM MSA-2 (Fig. 5c) and 1 nM diABZI (Suppl. Fig. S3). Interestingly, Western Blot analysis revealed the cleavage of gasdermin D, an indicator of pyroptosis, upon activation of monocytes with low dose MSA-2 (Fig. 5c) and low dose diABZI (Suppl. Fig. S3). Similarly, we detected cleaved gasdermin D in lysates of monocytes activated with the higher STING ligand concentrations, but only if TBK1 activation was simultaneously blocked by BX795 (Fig. 5c, Suppl. Fig. S3). Taken together, our results indicate that stimulation of pro-inflammatory IL-1β and TNF-α by STING ligands in human monocytes is associated with type I IFN signaling and massive apoptotic cell death, while induction of anti-inflammatory IL-10 leaves the majority of monocytes viable despite the observed cleavage of gasdermin D.

### Differential response pattern of M-CSF differentiated macrophages

**Figure 6:**
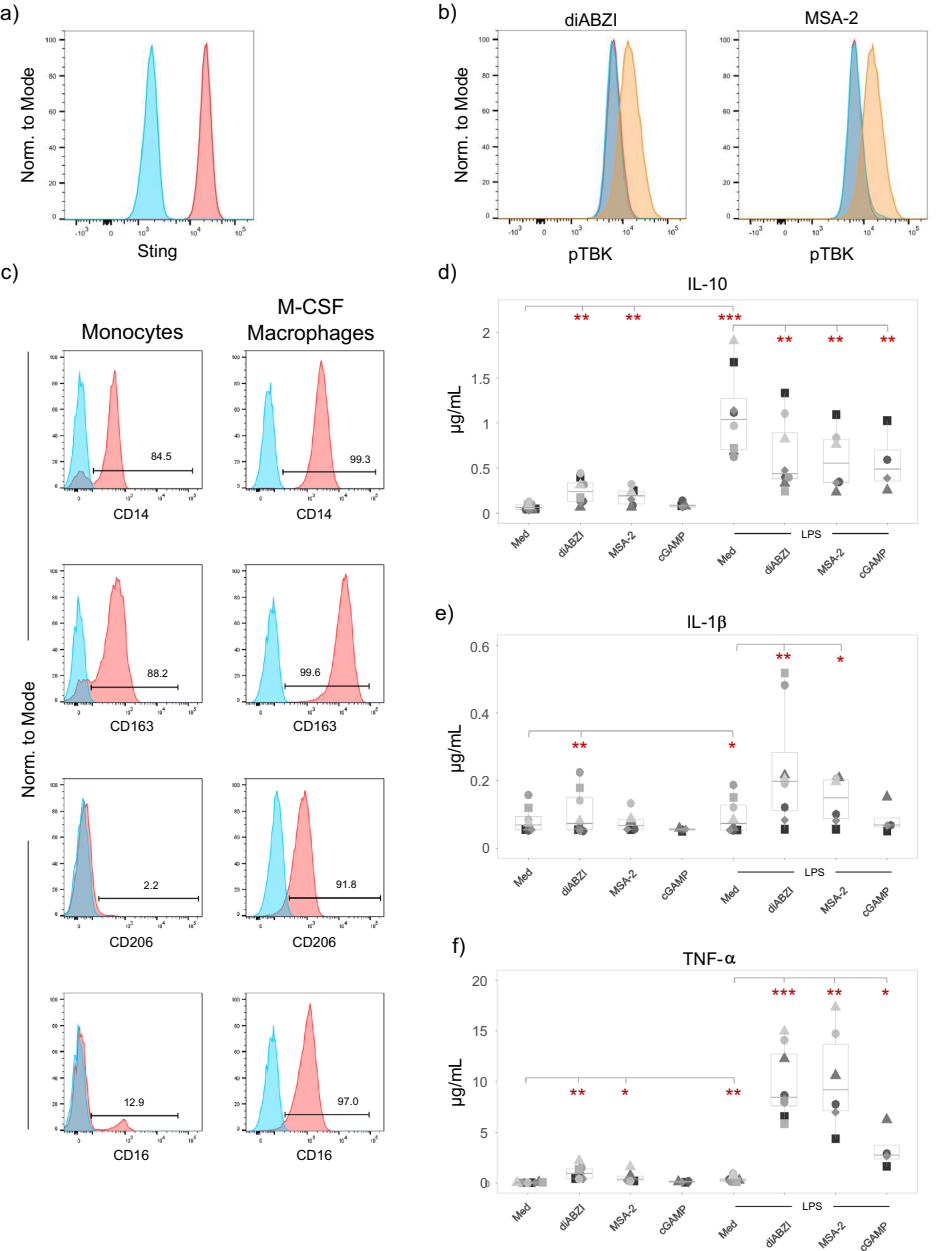
Responsiveness of M-CSF differentiated macrophages to STING ligands. Purified monocytes were cultured for 6 days in the presence of 50 ng/mL M-CSF. (**a**) M-CSF macrophages expressed STING as revealed by flow cytometry. Blue: FMO control; red: anti-STING-AF488. (**b**) M-CSF macrophages were stimulated with 100 nM diABZI or 25 µM MSA-2. After 60 min, phosphorylation of TBK1 was analyzed by flow cytometry using anti-pTBK1-PE antibody. Red: medium control; blue: lower dose of diABZI/MSA-2; beige: higher dose of diABZI/MSA-2. (**c**) Surface marker expression on paired samples of freshly isolated monocytes (left) and M-CSF macrophages (right). Blue: isotype controls; red: specific antibody. (**a**–**c**) Representative out of three experiments are shown. (**d**–**f**) M-CSF macrophages were cultured in medium only or were stimulated with 100 ng/mL LPS, 100 nM diABZI, 25 µM MSA-2 (left part), or the combination of LPS with STING ligands as indicated (right part). Supernatants were collected after 24 h, and cytokines were quantified by ELISA. (**d**) IL-10 (n = 5–8). (**e**) IL-1β (n = 6–8). (**f**) TNF-α (n = 3–6). Statistical significance between groups as indicated by the horizontal bars was calculated by paired Student’s test. Red asterisks indicate the level of significance between stimulated and unstimulated (medium) samples (left part) or between LPS plus STING ligands versus LPS only (right part). *p < 0.05, **p < 0.01.

Monocytes cultured in the presence of M-CSF acquire an anti-inflammatory phenotype^27^. Purified monocytes were cultured for 6 days with 50 ng/mL M-CSF, and M-CSF was added once again after 4 days. Such M-CSF macrophages expressed STING (Fig. 6a) and responded to the regular concentrations of diABZI (100 nM) and MSA-2 (25 µM) with rapid phosphorylation of TBK1 (Fig. 6b). In comparison to freshly isolated monocytes from the same donor, M-CSF macrophages exhibited enhanced expression of scavenger receptor CD163, mannose receptor CD206, as well CD16 (Fig. 6c). M-CSF macrophages were then stimulated with regular concentrations of diABZI (100 nM) and MSA-2 (25 µM) in the absence or presence of LPS, and cytokine levels were determined in comparison to similar experiments performed with freshly isolated monocytes (see Figs. 1, 2). diABZI and MSA-2 alone stimulated very low levels of IL-10, but also LPS was less efficient in comparison to freshly isolated monocytes (Fig. 6d). Interestingly, and in contrast to freshly isolated monocytes (Fig. 2a), the presence of STING ligands reduced but did not fully abolish the LPS-stimulated IL-10 secretion in M-CSF macrophages (Fig. 6d). In response to all tested stimuli, M-CSF macrophages were poor producers of IL-1β and secreted < 0.5 µg/mL, in comparison to > 5 µg/mL induced in freshly isolated monocytes by both LPS and STING ligands (Fig. 6e; compare with Fig. 1e). Finally, we observed remarkable co-stimulatory effects of LPS and the regular concentrations of diABZI and MSA-2 on TNF-α induction in M-CSF macrophages (Fig. 6f). Thus, while all three ligands were very weak stimuli on their own (< 2 µg/mL), potent co-stimulation of TNF-α secretion occurred when LPS was combined with 100 nM diABZI (6–15 µg/mL TNF-α) or with MSA-2 (5–17 µg/mL TNF-α). Co-stimulatory effects between LPS and STING ligands were also observed with freshly isolated monocytes (Fig. 2d), but here the STING ligands triggered TNF-α secretion already on their own (Fig. 1f).

## Discussion

In this study we discovered a previously unrecognized signal strength-dependent plasticity of human monocytes in response to STING activation. In line with previous reports, CDN as well as novel non-CDN STING ligands used at reported EC_50_ concentrations triggered the type I IFN signaling pathway as evidenced by TBK1 phosphorylation and secretion of IFN-β and IP-10. Moreover, these commonly used concentrations of STING ligands also stimulated massive production of pro-inflammatory cytokines IL-1β and TNF-α associated with apoptotic cell death, but no secretion of anti-inflammatory IL-10 and immunoregulatory IL-19. Strikingly, however, potent induction of IL-10 and IL-19 secretion was revealed by much lower (one- to two-log) concentrations of STING ligands.

CDN like 2′3′-cGAMP are the natural ligands for STING, produced by cytosolic cGAS in response to recognition of microbial or endogenous DNA which can originate from extracellular self-DNA or cell-intrinsic mitochondrial and genomic DNA^2,3,28,29^. cGAMP can be also released by tumor cells and activate neighboring cells following entry via bidirectional transporters like LRRC8A and LRRC8C/E heteromeric channels^16,30^. Additional CDN transporters have been identified^14,15^ and there is also evidence for the expression of an alternatively spliced STING isoform in the plasma cell membrane which can initiate immune responses by directly sensing extracellular cGAMP^17^. The expression of such transporters may vary among cell types and thus account for differential responses. Additional complexity is introduced by the report that adenosine-containing CDN like cGAMP can bind to the adenosine A2a receptor selectively expressed on the cell surface of human monocytes and induce apoptosis independently of STING^31,32^. The identification of highly potent novel non-CDN STING ligands which can easily enter into cells without the need of dedicated transporter molecules is a major step forward towards clinical application of STING ligands for instance as adjuvants in tumor immunotherapy^19,20,22,33^ or as anti-infective therapeutics in viral infections including Sars-CoV-2^23,24,34,35^. In our study we used the non-CDN STING ligands diABZI^19^ and MSA-2^21^ which we have previously shown to co-stimulate human γδ T-cell activation in a monocyte-dependent manner^36^.

In addition to stimulation of the type I IFN signaling pathway, STING ligands in the range of the commonly used EC_50_ concentrations also potently activate the inflammasome thereby leading to caspase-1 cleavage-dependent secretion of IL-1β and IL-18^4,9^. In contrast to murine monocytes, human monocytes activate an alternative inflammasome which allows them to secrete IL-1β without a priming step required for canonical NLRP3 activation^13^ and in the absence of K^+^ efflux and pyroptosis^8^. It was shown that STING signaling leads to apoptotic cell death and IL-1β secretion associated with K^+^ efflux upstream of NLRP3 activation in human monocytes^9^. We observed that purified monocytes rapidly die in response to those commonly used concentrations of STING ligands. Induction of cell death was associated with cleavage of caspase-3 and PARP-1 and thus displayed characteristics of apoptosis. Gaidt et al. reported that STING activation of human myeloid cells triggered a lysosomal death program where leakage of the lysosomal content including cathepsins into the cytosol induces cell death^9^. In our experiments, we detected the constitutive expression of the mature 28 kD form of cathepsin D and no alteration in response to STING activation. In previous studies, the maturation of the enzymatically active 32/28 kD form of cathepsin D has been shown to be mediated by ceramide^37^ which we have not addressed in our study. Taken together, our results observed with stimulation of purified monocytes with commonly used “normal” concentrations of STING ligands cGAMP, diABZI and MSA-2 are fully in line with previous research, i.e. our experiments confirm the reported potent stimulation of secretion of pro-inflammatory cytokines IL-1β and TNF-α associated with the simultaneous induction of massive apoptotic cell death^38^. Although Fas/CD95-dependent and spontaneous apoptosis of monocytes have been reported to induce the production of anti-inflammatory IL-10 in monocytes^39,40^, the STING-mediated apoptotic death did not trigger any IL-10 (and IL-19) secretion in our experiments, in line with the general assumption that the cGAS/STING pathway triggers type I IFN production via TBK1/IRF3 and pro-inflammatory cytokines via NF-κB^3^. Interestingly, diABZI and MSA-2 completely suppressed the LPS-induced secretion of IL-10 and IL-19, but only moderately reduced LPS-induced IL-1β and in fact co-stimulated LPS-induced TNF-α secretion (Fig. 2). It appears that STING ligand-induced initiation of cell death also shuts down the capacity to secrete IL-10 in response to LPS but does not prevent LPS-stimulated secretion of IL-1β and TNF-α. Cross-talk between innate immune sensors has been observed in other model systems, although the role of cell death induction has not been thoroughly addressed. Similar to our results with STING ligands, TLR7/8 ligand Resiquimod was also shown to inhibit IL-10 production in human monocytes^26^. In other studies, activation of cGAS/STING was found to inhibit TLR9-mediated production of type I IFN in human plasmacytoid dendritic cells^41^ but to enhance LPS-induced type I IFN pathway activation in monocytic THP-1 cells and murine bone marrow derived dendritic cells^42^.

Remarkably different results were obtained when we used two-log (diABZI) and one-log (MSA-2) lower concentrations of STING ligands. In this setting, we observed potent induction of IL-10 and IL-19 but strongly reduced secretion of IL-1β and TNF-α using 1 nM diABZI and 2.5 μM MSA-2. Importantly, such low concentrations did neither induce cell death nor cleavage of caspase-3 and PARP-1, and no TBK1 activation as revealed by Western blot. Furthermore, the low diABZI and MSA-2 concentrations also did not stimulate any IFN-β secretion, in contrast to the regular 100 nM diABZI and 2.5 μM MSA-2 concentrations. However, IL-10 secretion by low dose STING ligands was prevented by BX795 which inhibits the catalytic activity of TBK1 suggesting that low level functional activity of TBK1 might still be required for IL-10 induction. In fact, such low level TBK1 phosphorylation was observed by flow cytometry. Interestingly, only at low but not high concentrations of diABZI and MSA-2 we also observed cleavage of gasdermin D which is the downstream effector of pyroptosis^43,44^. Surprisingly, however, cleaved gasdermin D was also specifically detected in lysates of monocytes activated with high STING ligand concentration (100 nM diABZI, 2.5 μM MSA-2) in the presence of the TBK1 inhibitor BX795. While the cleavage of gasdermin D is commonly considered an indicator of pyroptosis^44^, it is known that pore formation (and thus cell death) requires the additional activity of the Ragulator-Rag complex which promotes oligomerization of cleaved gasdermin in the plasma membrane^45^. This may explain the appearance of cleaved gasdermin D despite the lack of significant cell death in monocytes stimulated with the low STING ligand concentrations, and thus underscores the importance of non-lytic functions of gasdermin D^46^.

In addition to other immune cells like Th2 T cells, monocytes are well characterized producers of IL-10, and multiple microbial stimuli can trigger IL-10 production in monocytes^47–49^. IL-10 is regulated by transcription factors including Blimp-1 and c-Maf and by epigenetic mechanisms^50–52^. IL-10 is a major anti-inflammatory cytokine with multiple functions in the immune system but also in epithelial repair^53^. Therefore, there is major interest to characterize in detail the regulation of IL-10 expression. Induction of IL-10 and related immunoregulatory cytokines like IL-19 by activation of the cGAS/STING pathway has not been reported before for human monocytes. In fact, there is only very limited information available under which conditions the cGAS/STING pathway might also initiate anti-inflammatory responses. Comparing wild-type, IL-10 deficient and STING-deficient mice, Ahn et al. obtained evidence for STING-dependent production of IL-10 stimulated by commensal bacteria to control gut homeostasis^54^. We verified the induction of IL-10 by low dose STING ligands as measured by ELISA also by intracellular flow cytometry and qRT-PCR. In line with a role of Blimp-1, we detected similar levels of *PRDM1* induction by low dose STING ligands and the standard concentration of LPS. In addition to IL-10, we also measured IL-19 which is an immunoregulatory cytokine involved in the resolution of inflammation and a member of the extended IL-10 family^55,56^. The concentration-dependent pattern of induction by STING ligands was identical for IL-10 and IL-19, with strong induction by low concentrations (1 nM diABZI, 2.5 μM MSA-2) and absolutely no induction by regular concentrations (100 nM diABZI, 25 μM MSA-2).

The signal strength-dependent plasticity in response to STING activation described here is not unique for human monocytes. It has been reported that murine T cells have an intrinsically higher expression of STING when compared to bone marrow-derived macrophages, and readily undergo apoptosis in the presence of murine STING activators CMA or DMXAA^6^. By contrast, bone marrow-derived macrophages have very low STING expression and do not undergo apoptosis in response to STING ligands unless transduced with an inducible STING construct which allows controlled increase of STING expression^6^. In previous studies, we also observed differential effects of STING ligands on resting versus activated T cells. While diABZI and MSA-2 co-stimulated activation of human γδ T cells within PBMC, both STING ligands also triggered massive cell death in short-term γδ T-cell lines expanded for 14 days in vitro^36^. In line with a stage of differentiation dependent plasticity of the STING responsiveness of myeloid cells, we also noticed significant differences between freshly isolated monocytes and M-CSF differentiated macrophages. As expected, M-CSF macrophages expressed higher levels of CD163, CD206 and CD16. Although these cells expressed STING and responded to 100 nM diABZI and 25 μM MSA-2 with measurable TBK1 phosphorylation, there was very little induction of IL-1β and TNF-α in response to STING ligands but also in response to LPS. M-CSF macrophages were less susceptible to STING-induced cell death and also secreted low amounts of IL-10 at these concentrations. In striking contrast to freshly isolated monocytes, IL-10 stimulated by LPS was somewhat reduced but not completely inhibited by the STING ligands. While M-CSF macrophages produced drastically less IL-1β compared to fresh monocytes, we observed a very strong co-stimulatory effect of the STING ligands on the LPS-induced TNF-α secretion. Overall, our results emphasize the scenario that the result of STING activation in a given cell system will depend on (at least) three parameters, i.e. (1) the cell type, (2) the stage of differentiation, and (3) the available concentration of STING ligands. Additional complexity is envisaged when extrapolating in vitro results to in vivo conditions where the three-dimensional tissue and its cellular composition have a major impact.

We have characterized experimental conditions where the cytokine spectrum of human monocytes in response to STING ligands can be switched from pro-inflammatory to anti-inflammatory. We are fully aware of the limitations of this in vitro study as we do not yet have evidence that such a mechanism is at work also in vivo. However, it can be easily imagined that different ranges of endogenous DNA concentrations resulting from differential levels of cellular stress or (tumor) cell death might also trigger anti- versus pro-inflammatory cytokines in vivo. In this respect, it can be speculated that low levels of tumor-derived DNA might favor an anti-inflammatory (and thus perhaps pro-tumorigenic) phenotype of monocytes present in the tumor micromilieu, whereas higher concentrations of endogenous DNA or exogenously applied STING ligands will activate the pro-inflammatory phenotype associated with death of the local monocytes.

## Concluding remarks

We demonstrate that commonly used concentrations of STING ligands stimulate pro-inflammatory cytokines and cell death in human monocytes, whereas substantially lower concentrations stimulate anti-inflammatory cytokines and no cell death. The newly identified signal strength-dependent plasticity reveals the versatility of human monocytes in response to activation of the STING pathway which might be an important parameter also in the context of clinical application of STING ligands.

## Supplementary Information

Supplementary Figures.

Supplementary Information.

## Data Availability

The datasets generated during and/or analyzed during the current study have not been submitted to public repository but are available from the corresponding author on reasonable request.
