# Supplementary material for: Signal strength of STING activation determines cytokine plasticity and cell death in human monocytes
Source: Sci Rep. 2022 Oct 24;12:17827. doi: 10.1038/s41598-022-20519-7 (PMC9590392; doi:10.1038/s41598-022-20519-7)
Supplement: Supplementary file 1 — Supplementary Figures. [file 41598_2022_20519_MOESM1_ESM.pdf]

Supplemental Figures 1 - 3

Signal strength of STING activation determines cytokine plasticity and cell death in human monocytes

Dieter Kabelitz, Michal Zarobkiewicz, Michelle Heib, Ruben Serrano, Monika Kunz, Guranda Chitadze, Dieter Adam, Christian Peters

Supplemental Figure S1

Modulation of monocyte culture morphology by STING ligands

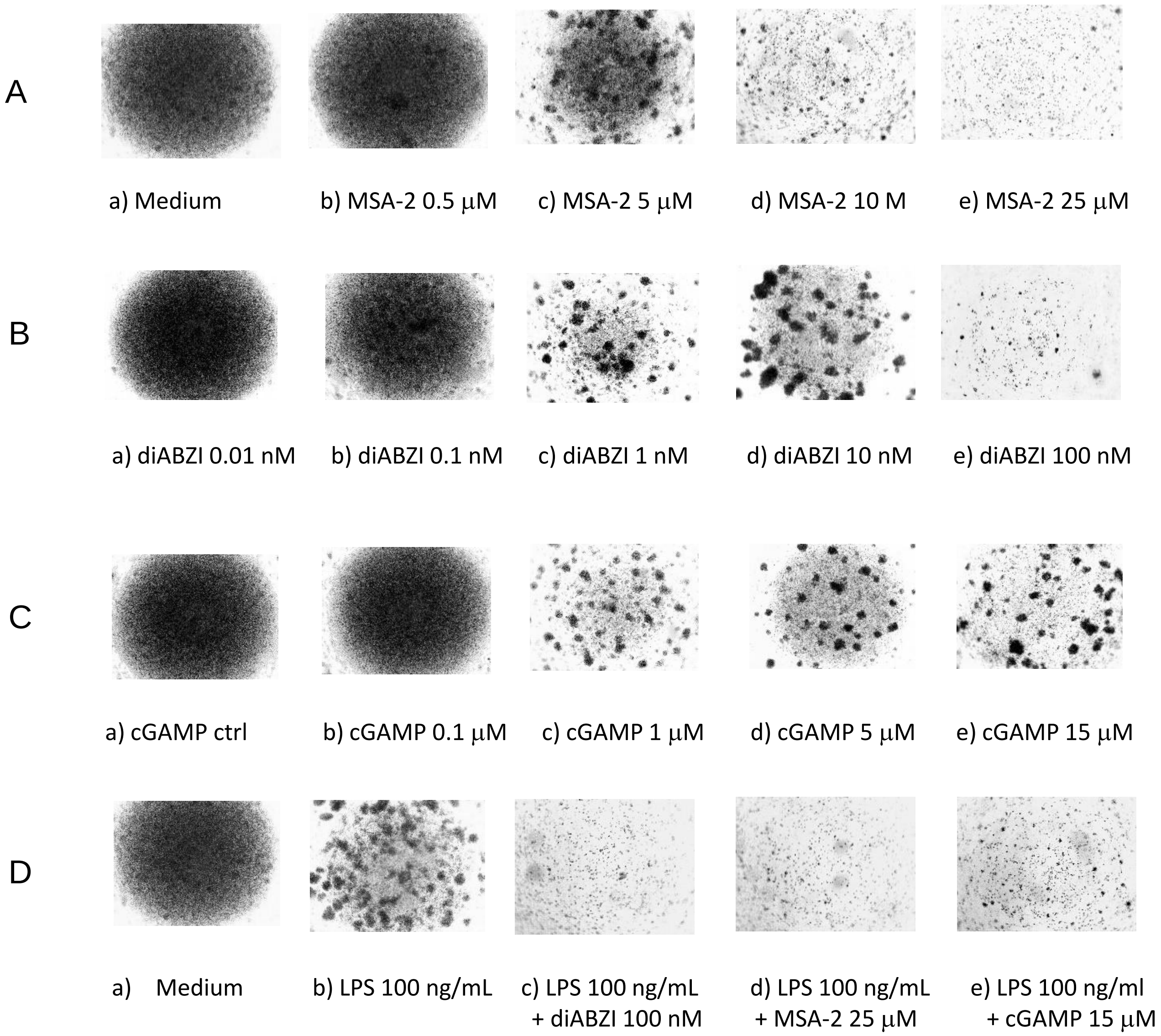

100.000 purified monocytes per well were cultured in round-bottom 96-well micro-titer plates in the absence (medium) or presence of the indicated concentrations of STING ligands and/or LPS. Pictures were taken after 48 h with a Leitz Axiovert microscope at magnification x100.

Supplemental Figure S2

Responsiveness of monocytes from donors with high spontaneous IL-10 secretion to STING ligands

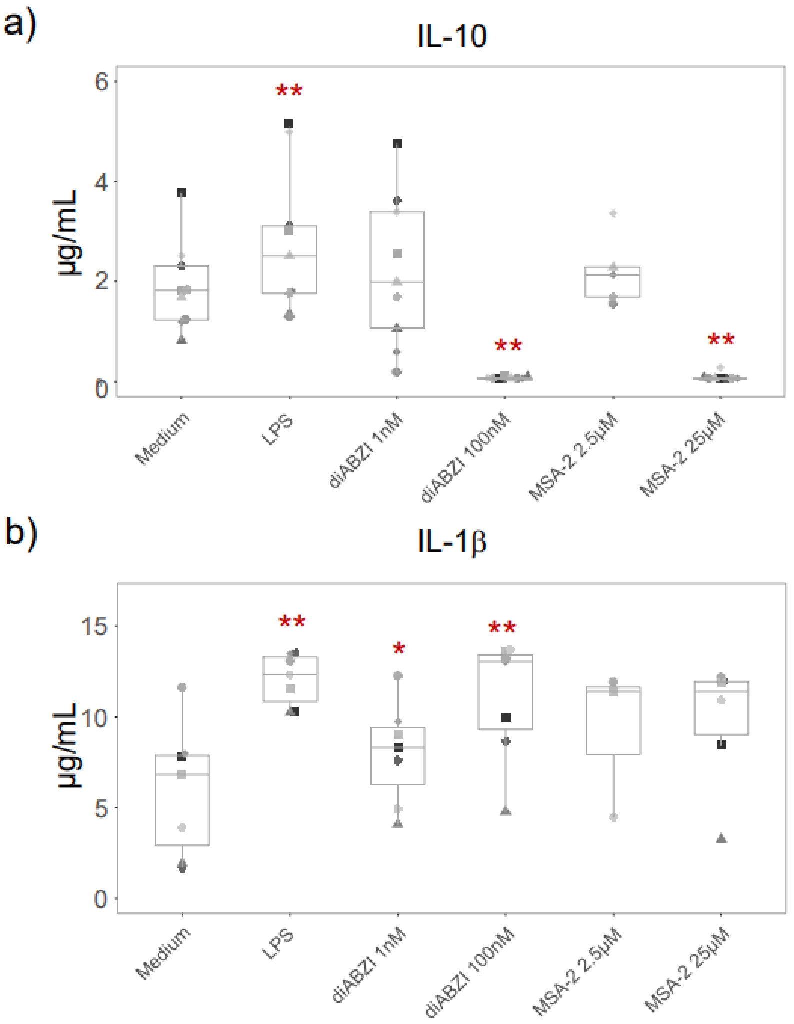

Purified monocytes from donors with spontaneous secretion of > 1 µg/mL were stimulated with 100 ng/mL LPS, 1 nM/100 nM diABZI, or 2.5 µM/25 µM MSA-2 as indicated. Cytokines were quantified by ELISA in supernatants after 24 h. Each donor is identified by identical symbol and grey tone. a) IL-10 (n = 5-8). b) IL-1β (n = 3-6). Statistical significance of stimulated samples versus medium control was calculated by paired Student's t-test. * $p < 0.05$, ** $p < 0.01$.

Supplemental Figure S3

Western blot analysis of monocytes activated by STING ligands

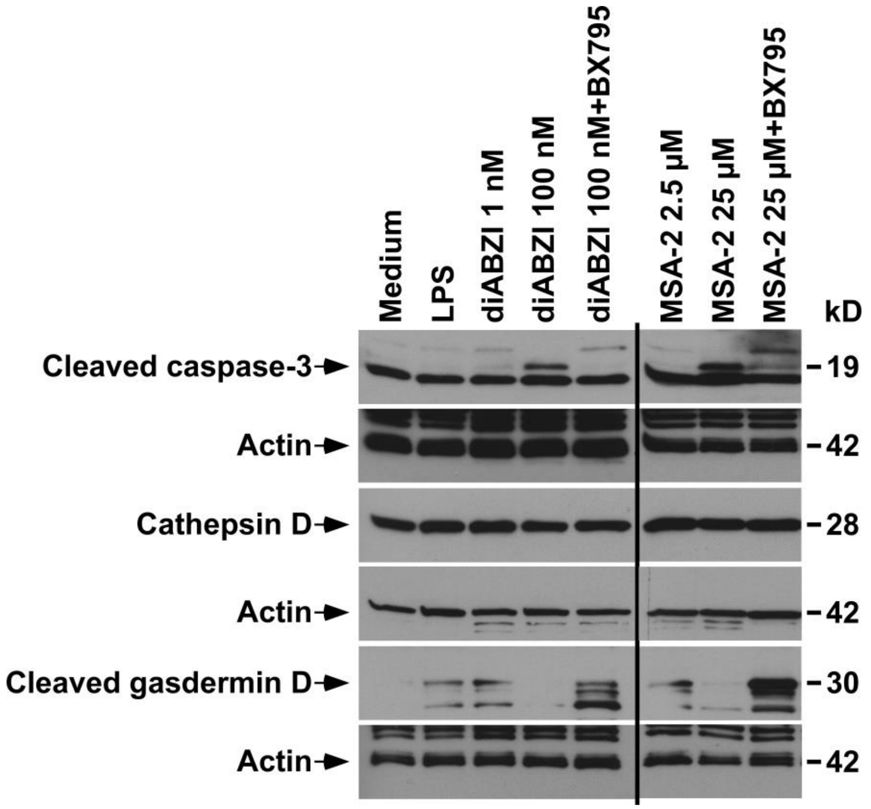

Purified monocytes were stimulated for 7 h with 100 ng/mL LPS or the indicated concentrations of STING ligands in the absence or presence of 1 μM BX795.
