# Supplementary material for: Signal strength of STING activation determines cytokine plasticity and cell death in human monocytes
Source: Sci Rep. 2022 Oct 24;12:17827. doi: 10.1038/s41598-022-20519-7 (PMC9590392; doi:10.1038/s41598-022-20519-7)

Uncropped Western Blots for Figure 1b (STING, TBK1, pTBK1)

White rectangles indicate areas shown in Figure 1b, red dashed lines separate individual membrane slices.

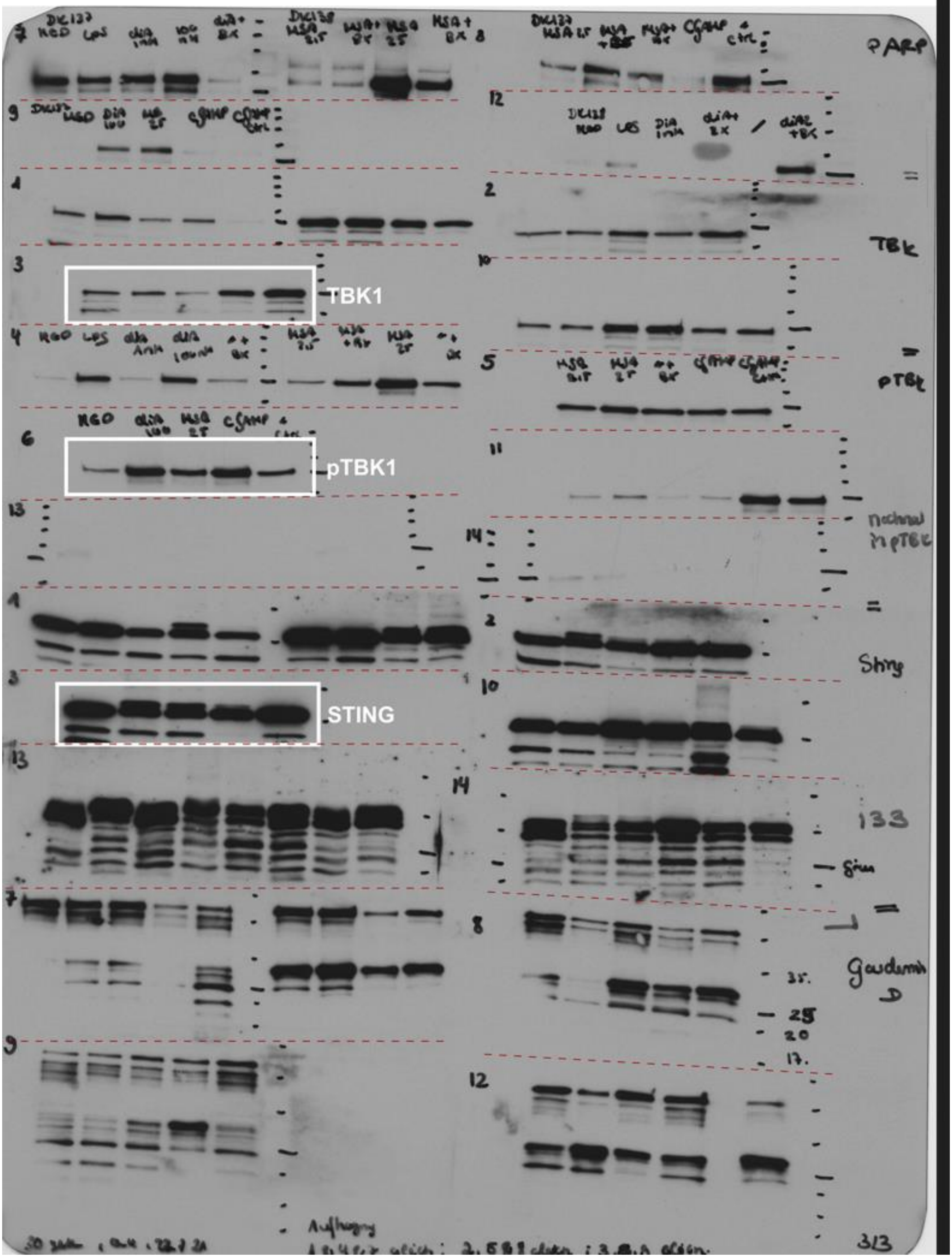

Uncropped Western Blots for Figure 1b (Actin for STING, TBK1)

A white rectangle indicates the area shown in Figure 1b, red dashed lines separate individual membrane slices.

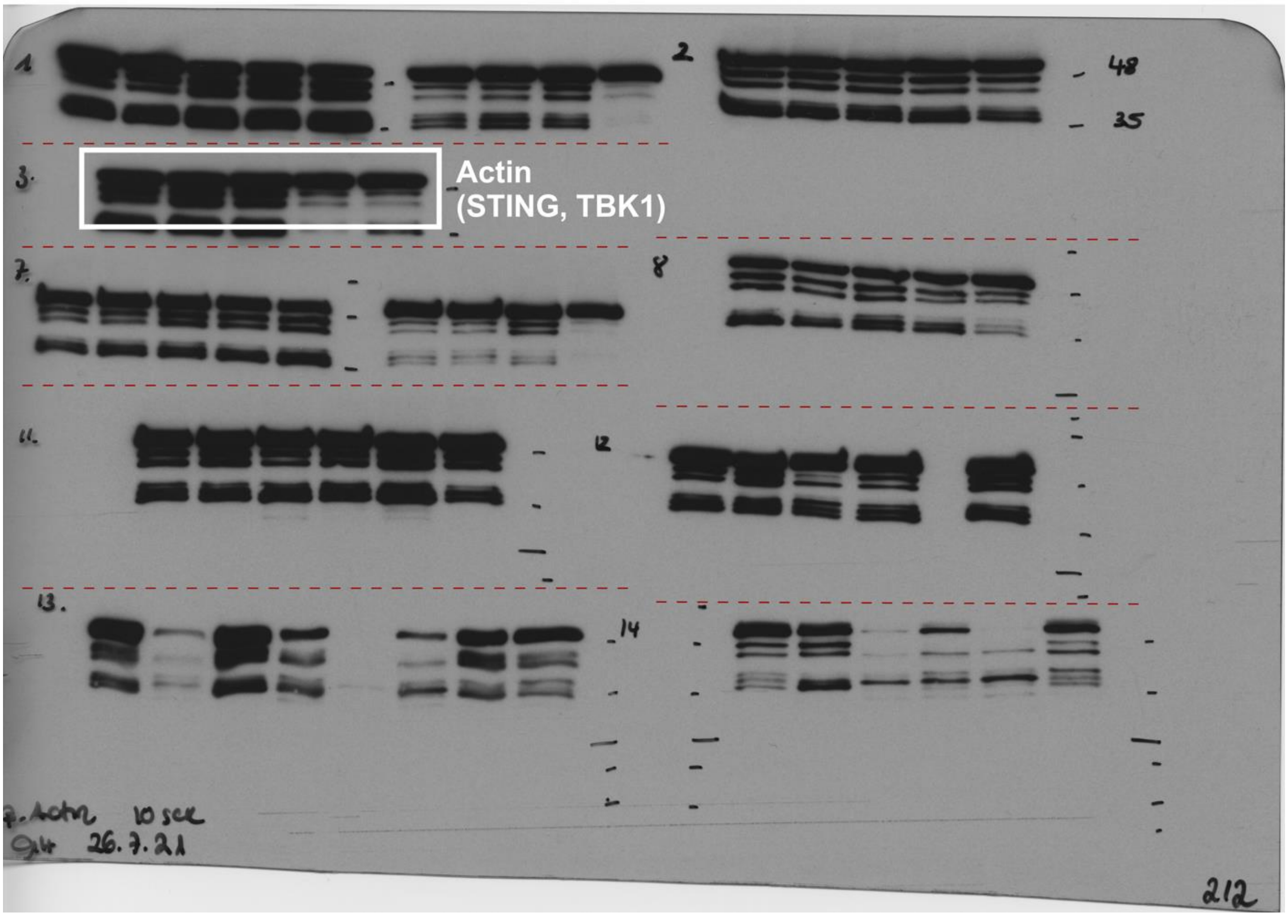

Uncropped Western Blots for Figure 1b (Actin for pTBK1)

A white rectangle indicates the area shown in Figure 1b, red dashed lines separate individual membrane slices.

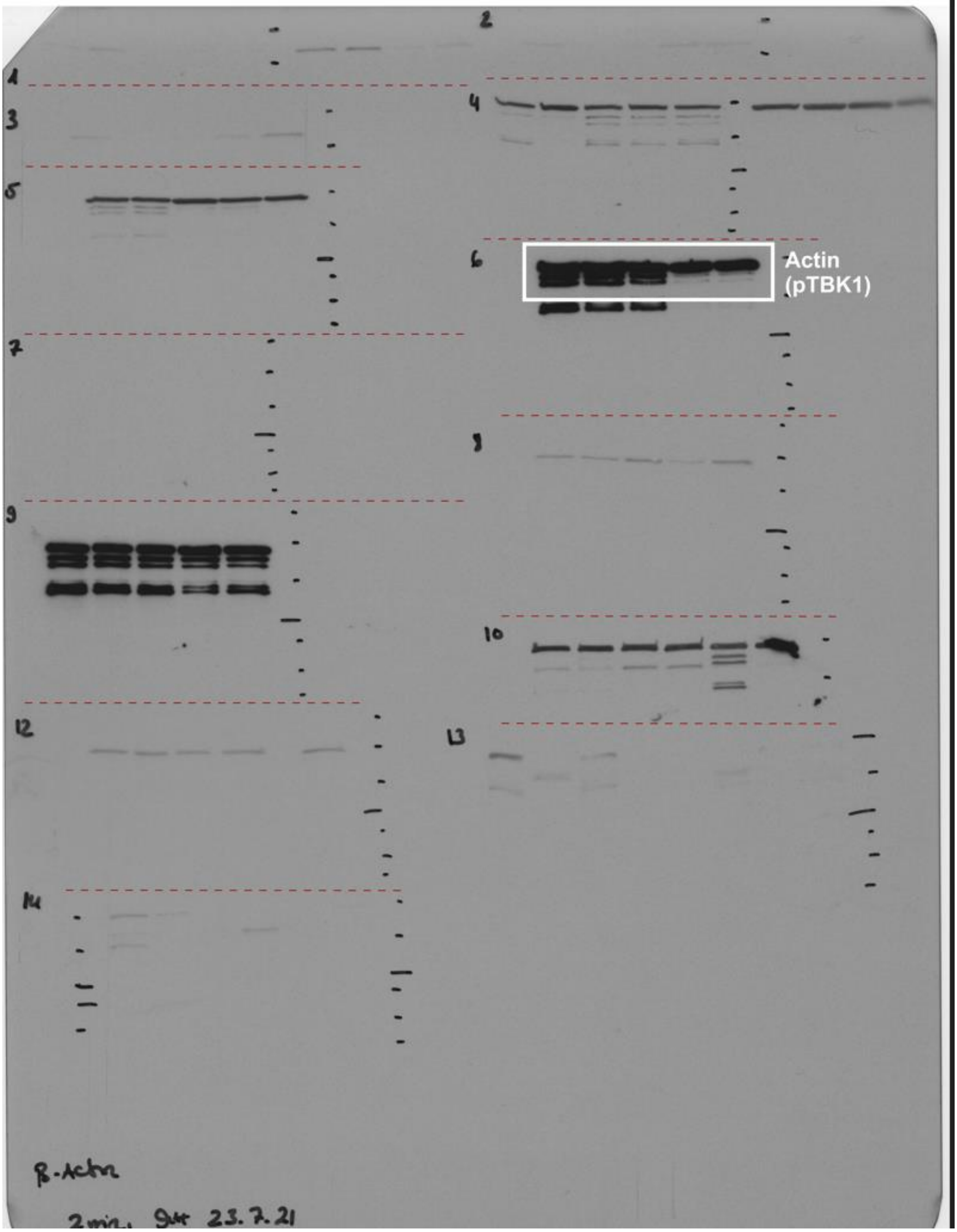

Uncropped Western Blots for Figure 4b (TBK1, pTBK1)

White rectangles indicate areas shown in Figure 4b, red dashed lines separate individual membrane slices.

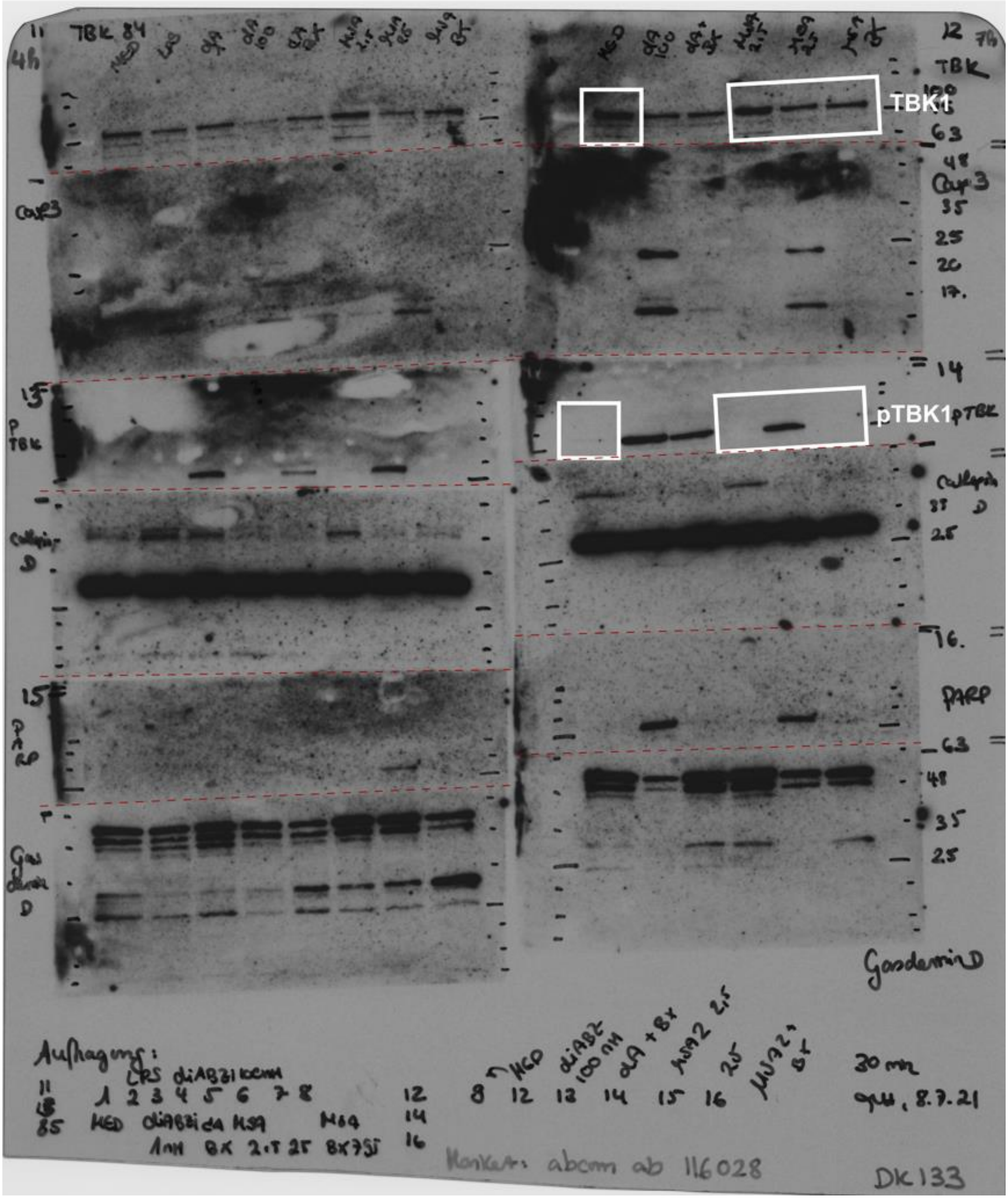

Uncropped Western Blots for Figure 4b (Actin for TBK1, pTBK1)

White rectangles indicate areas shown in Figure 4b, red dashed lines separate individual membrane slices.

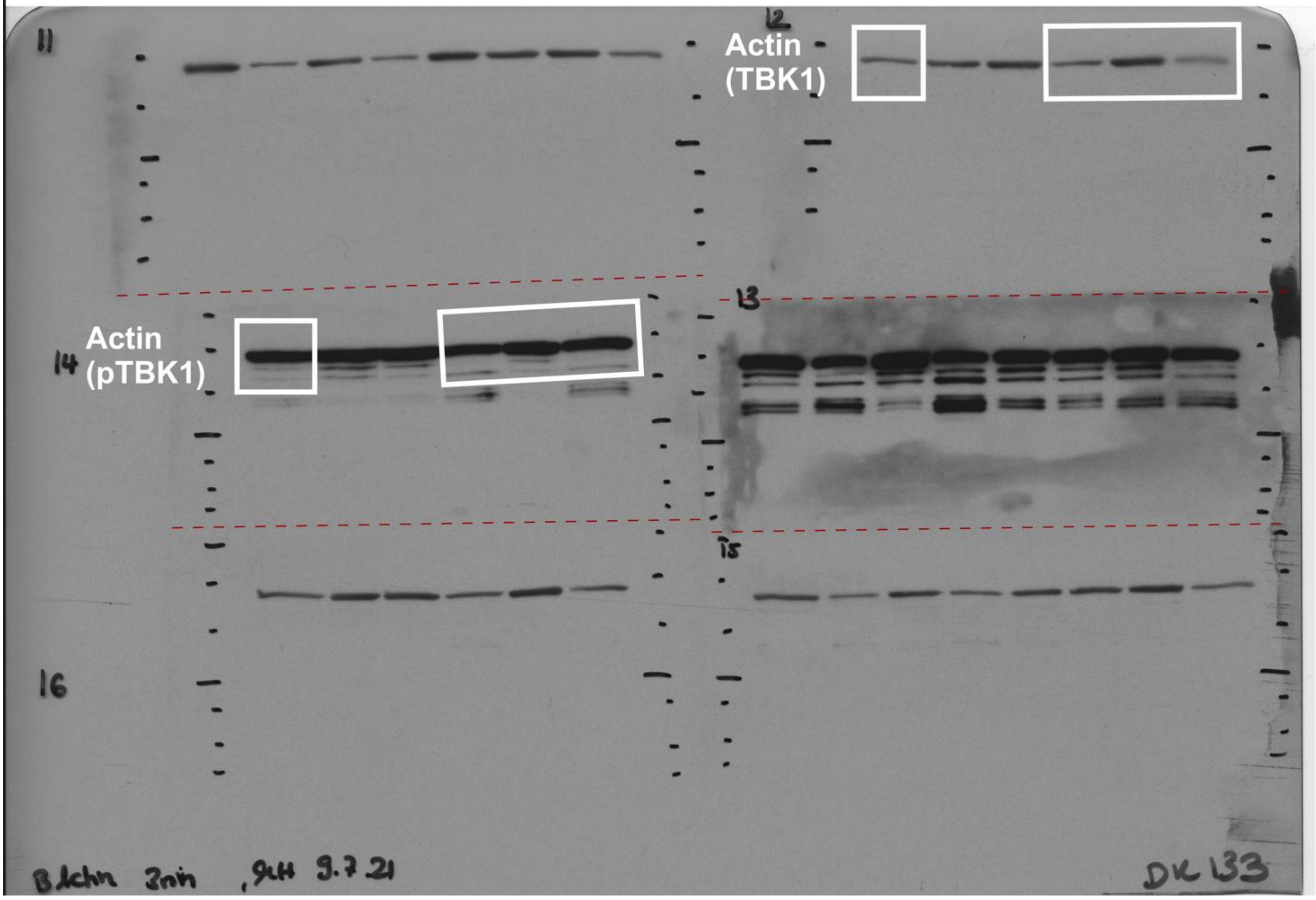

Uncropped Western Blots for Figure 5c (cleaved caspase-3, cathepsin D, cleaved PARP-1, cleaved gasdermin D)

White rectangles indicate areas shown in Figure 5c, red dashed lines separate individual membrane slices.

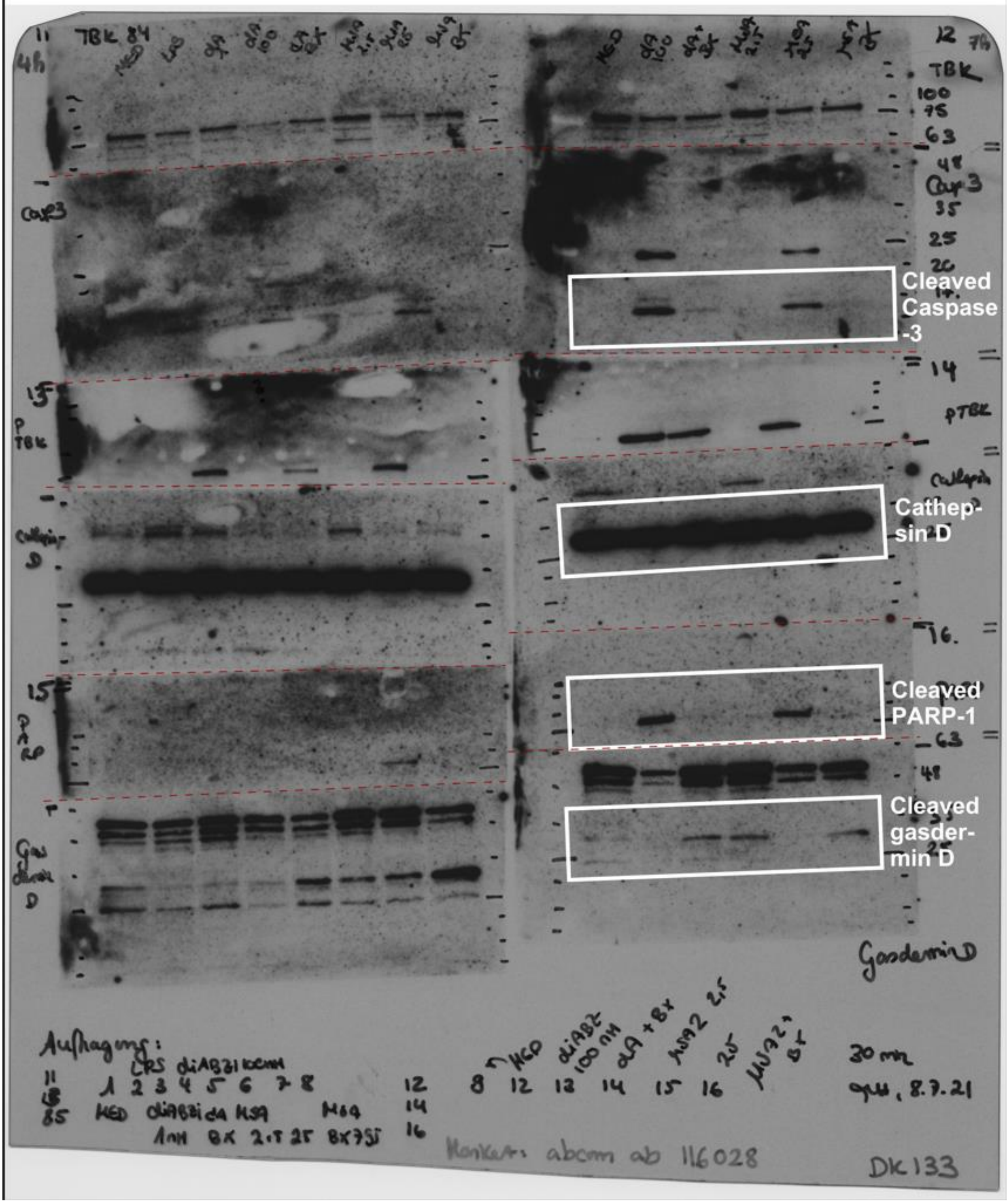

Uncropped Western Blots for Figure 5c (Actin for cleaved caspase-3, cathepsin D, cleaved PARP-1, cleaved gasdermin D)

White rectangles indicate areas shown in Figure 5c, red dashed lines separate individual membrane slices.

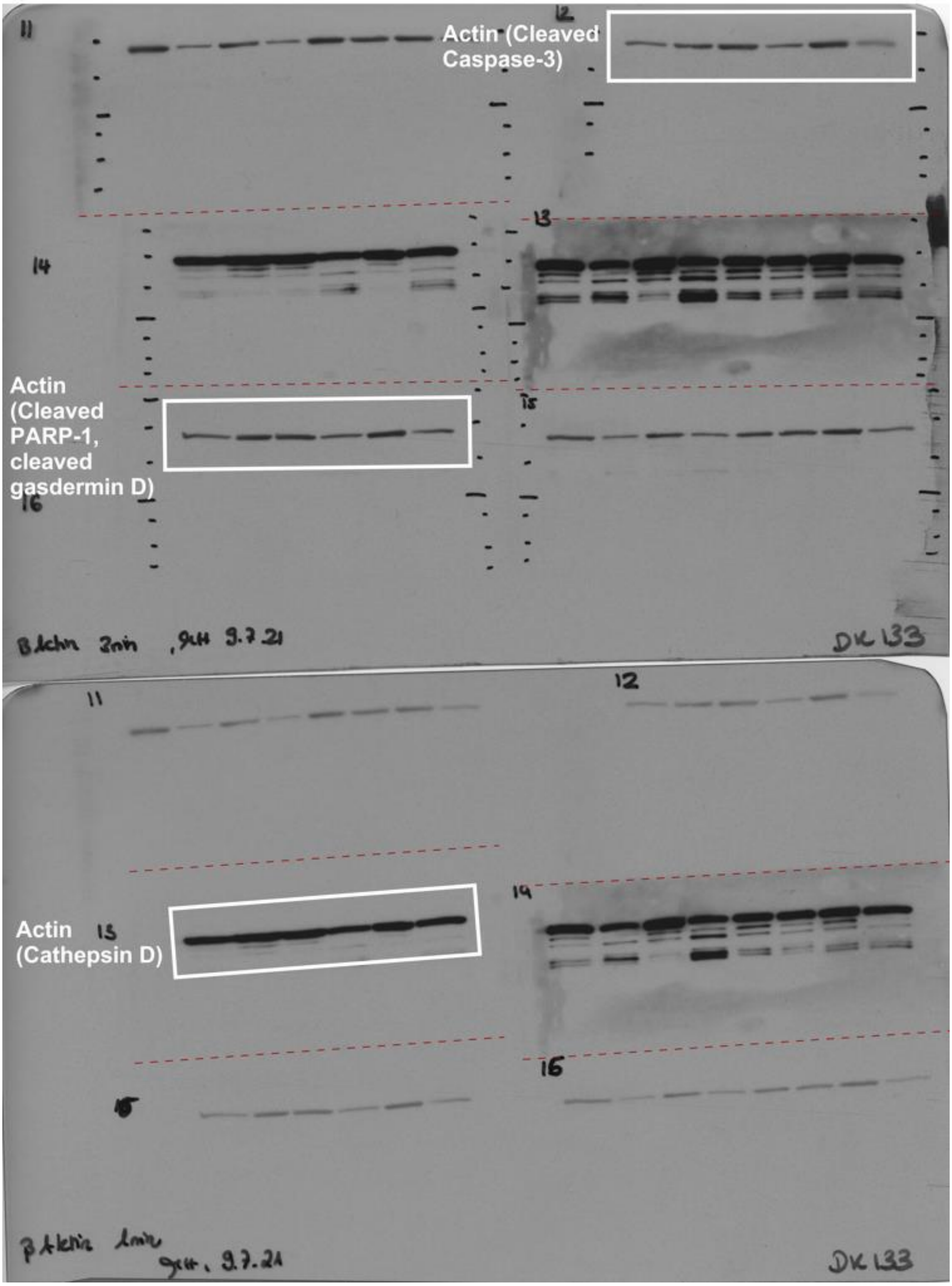

Uncropped Western Blots for Supplemental Figure S3 (Actin for cathepsin D

White rectangles indicate areas shown in Supplemental Figure S3, red dashed lines separate individual membrane slices.

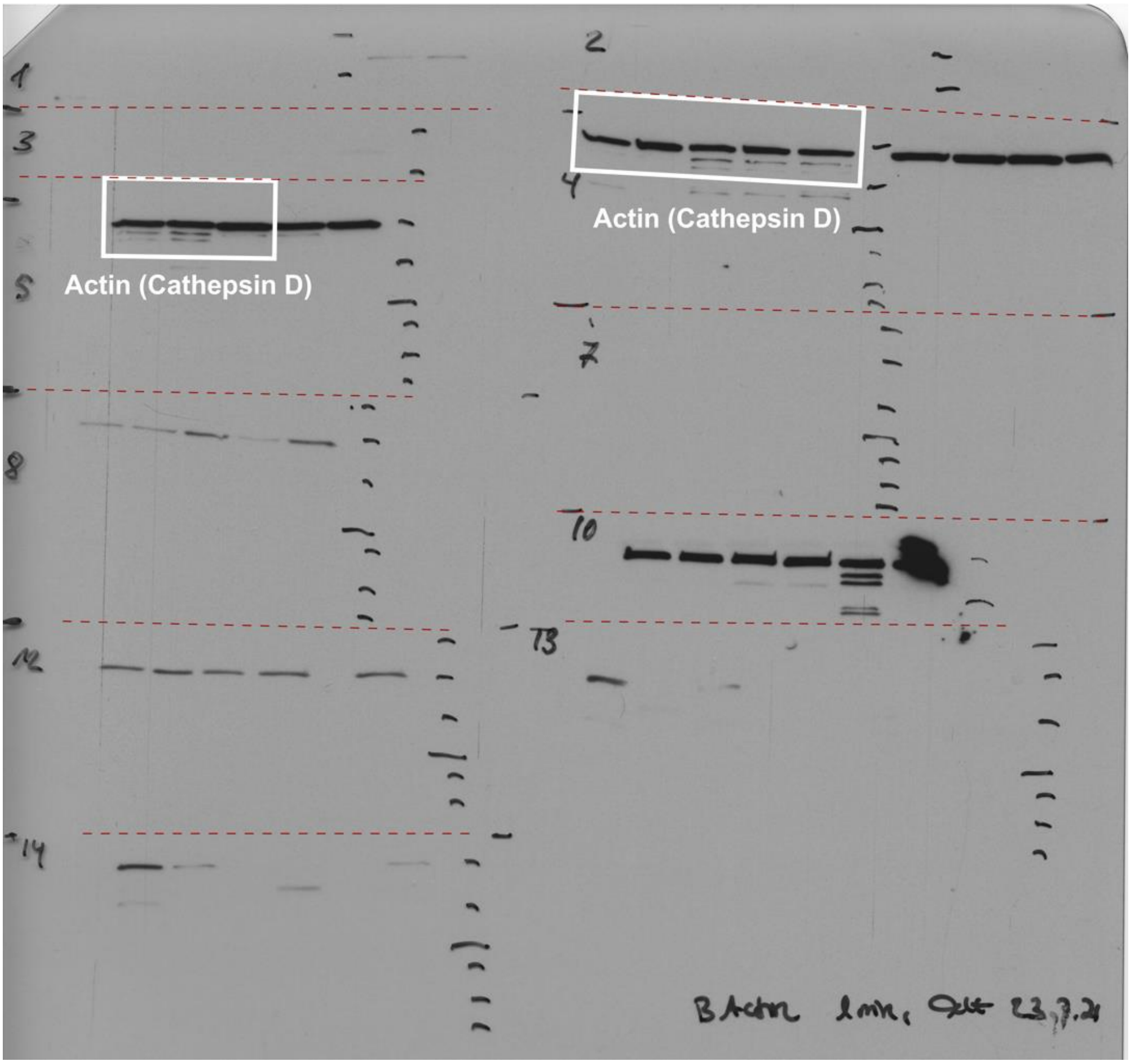

Uncropped Western Blots for Supplemental Figure S3 (Cleaved gasdermin D)

White rectangles indicate areas shown in Supplemental Figure S3, red dashed lines separate individual membrane slices.

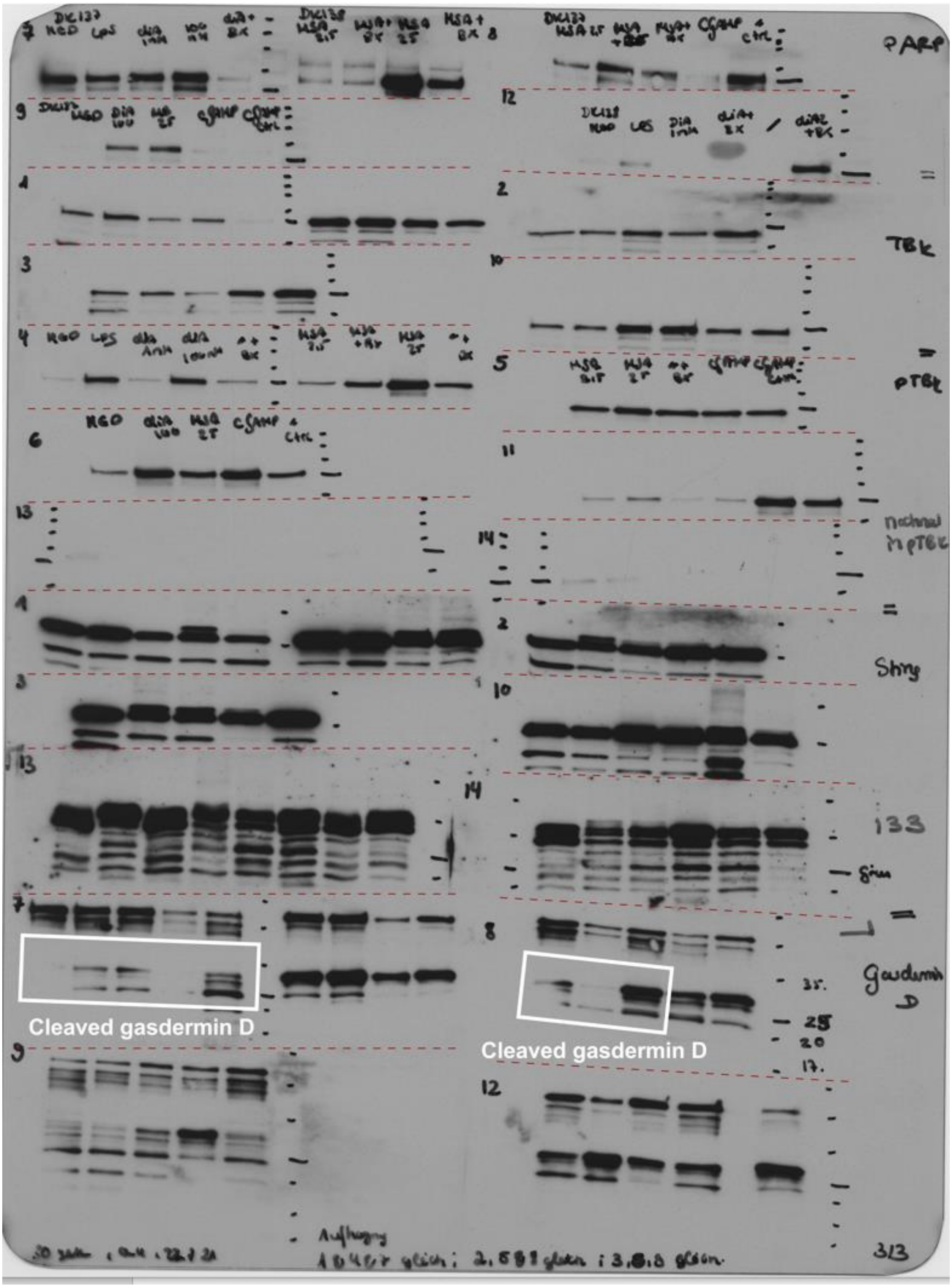

Uncropped Western Blots for Supplemental Figure S3 (Actin for cleaved caspase-3, cleaved gasdermin D)

White rectangles indicate areas shown in Supplemental Figure S3, red dashed lines separate individual membrane slices.

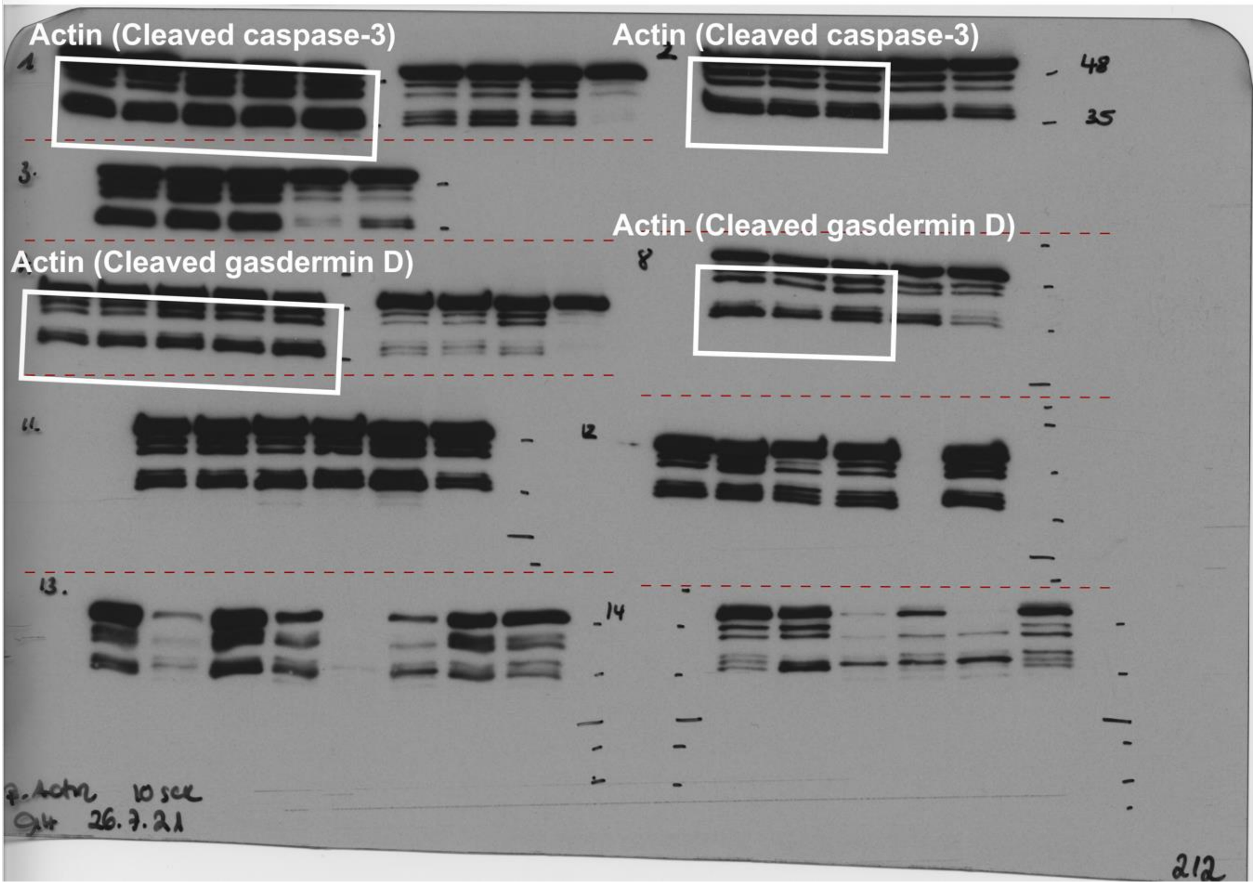

Uncropped Western Blots for Supplemental Figure S3 (Actin for cathepsin D

White rectangles indicate areas shown in Supplemental Figure S3, red dashed lines separate individual membrane slices.

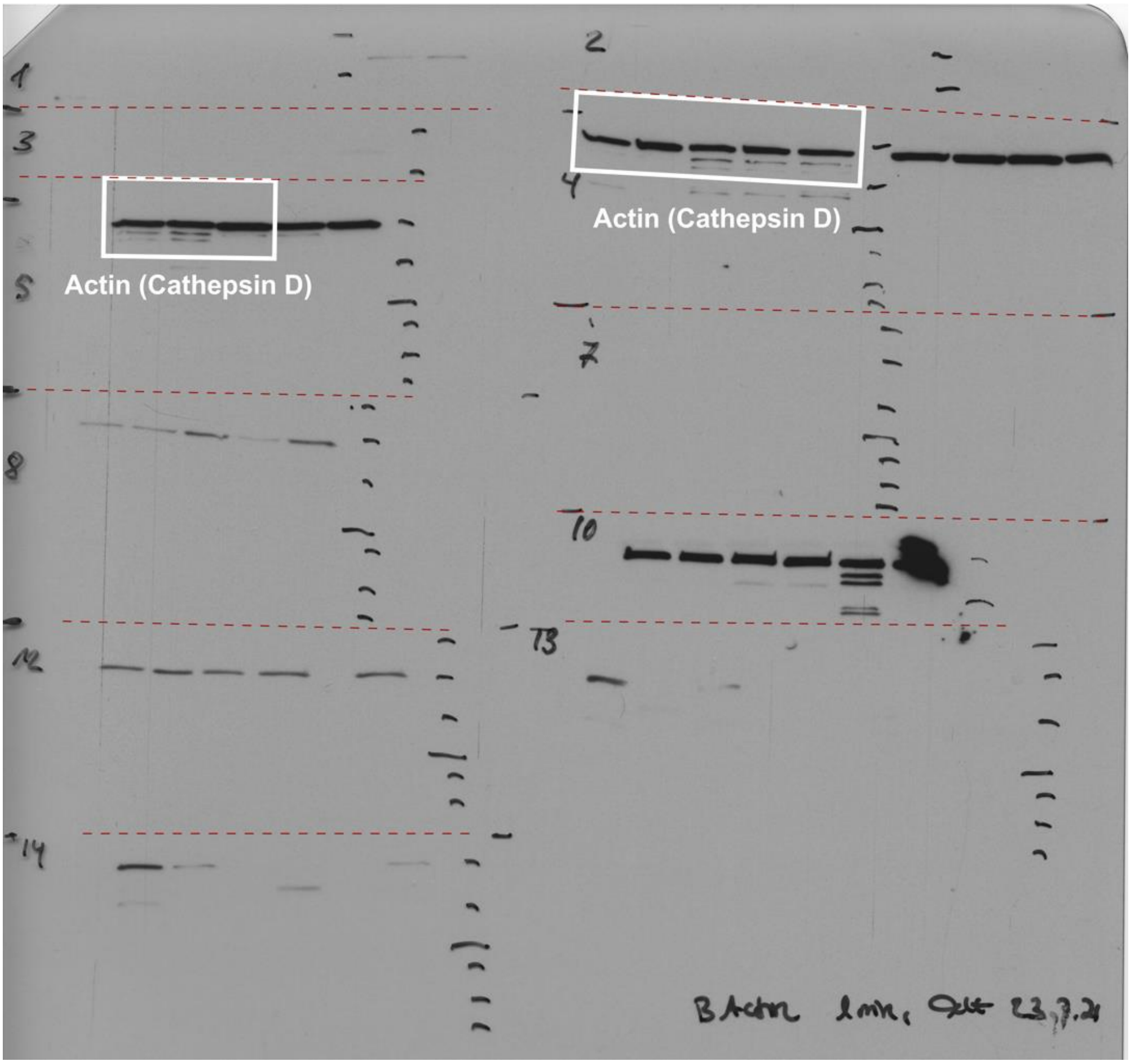

Supplement: Supplementary file 2 — Supplementary Information. [file 41598_2022_20519_MOESM2_ESM.pdf]
